# Long‐term shifts in the seasonal abundance of adult *Culicoides* biting midges and their impact on potential arbovirus outbreaks

**DOI:** 10.1111/1365-2664.13415

**Published:** 2019-05-29

**Authors:** Christopher J. Sanders, Chris R. Shortall, Marion England, Richard Harrington, Beth Purse, Laura Burgin, Simon Carpenter, Simon Gubbins

**Affiliations:** ^1^ The Pirbright Institute Pirbright UK; ^2^ Rothamsted Research Harpenden UK; ^3^ Centre for Ecology and Hydrology Wallingford UK; ^4^ The Met Office Exeter UK

**Keywords:** arbovirus, biting midges, bluetongue, climate change, long‐term data, Schmallenberg, transmission season, vector‐borne disease

## Abstract

1. Surveillance of adult *Culicoides* biting midge flight activity is used as an applied ecological method to guide the management of arbovirus incursions on livestock production in Europe and Australia.
2. To date the impact of changes in the phenology of adult vector activity on arbovirus transmission has not been defined. We investigated this at two sites in the UK, identifying 150,000 *Culicoides* biting midges taken from 2867 collections over a nearly 40 year timescale.
3. Whilst we recorded no change in seasonal activity at one site, shifts in first adult appearance and last adult appearance increased the seasonal activity period of *Culicoides* species at the other site by 40 days over the time period.
4. Lengthening of the adult activity season was driven by an increase in abundance of *Culicoides* and correlated with local increases in temperature and precipitation. This diversity in responses poses significant challenges for predicting future transmission and overwintering risk.
5. *Policy implications.* Our analysis not only shows a dramatic and consistent increase in the adult active period of *Culicoides* biting midges, but also that this varies significantly between sites. This suggests broad‐scale analyses alone are insufficient to understand the potential impacts of changes in climate on arbovirus vector populations. Understanding the impact of climate change on adult *Culicoides* seasonality and transmission of arboviruses requires the context of changes in a range of other local ecological drivers.

## 1 INTRODUCTION

Arthropod‐borne viruses (arboviruses) include some of the most important emerging and re‐emerging pathogens of humans, livestock, and wildlife worldwide (Gould, Pettersson, Higgs, Charrel, & Lamballerie, 2017; Liang, Gao, & Gould, 2015; Weaver & Reisen, 2010). The seasonal incidence and abundance of arthropod vectors capable of transmitting arboviruses, alongside environmental temperatures enabling replication of the arbovirus in these vectors, are key determinants of the timing, intensity, and spread of outbreaks (Lafferty, 2009; Purse, Carpenter, Venter, Bellis, & Mullens, 2015; Rogers & Randolph, 2006). The potential influence of climate on the distribution and dynamics of both arthropod vectors and the arboviruses they transmit, both historically and in prediction under future climate change scenarios, has been a key area of debate (Gould & Higgs, 2009; Kovats, Campbell‐Lendrum, McMichael, Woodward, & Cox, 2001; Lafferty, 2009; Tabachnick, 2016).

The unprecedented incursion and establishment of multiple strains and species of arboviruses transmitted by *Culicoides* biting midges (Diptera: Ceratopogonidae) across Western Europe is a spectacular example of a shift in global pathogen distribution and has been suggested to be driven by changing climate (Elbers, Koenraadt, & Meiswinkel, 2015; Gubbins, Carpenter, Baylis, Wood, & Mellor, 2008; Purse et al., 2005). This hypothesis is underpinned by the fact that *Culicoides* are among the vector groups most likely to be affected by changes in temperature and precipitation, being small‐bodied (<1.5 mm body length) and entirely reliant on the presence of suitable semi‐aquatic habitats for larval development (Purse et al., 2005). Competing hypotheses for these incursions, including changes in livestock husbandry, distribution, and abundance, have previously been discounted (Carpenter, Wilson, & Mellor, 2009; MacLachlan & Guthrie, 2010; Purse et al., 2005).

Insect phenology is a strong biological indicator for the impacts of climate change (Cleland, Chuine, Menzel, Mooney, & Schwartz, 2007; Forrest & Miller‐Rushing, 2010; Root et al., 2003) and plays a key role in our understanding potential changes in ecosystem processes (Diez et al., 2012; Rafferty, CaraDonna, Burkle, Iler, & Bronstein, 2013). The magnitude and direction of response to shifts in climate varies between species, often depending on ecological traits, but there is a general trend for spring‐time events such as adult emergence, to take place earlier in response to shortening winters in temperate regions (Menzel et al., 2006; Thackeray et al., 2016, 2010). The seasonal timing of interactions between vectors, pathogens, and hosts may also shift in ways that may promote or hinder pathogen transmission (Altizer et al., 2006).

Surveillance of adult *Culicoides* flight periods is currently used in Europe and Australia as a means of demarcating a period during which the probability of arbovirus transmission is negligible (Searle et al., 2014). In northern Europe this enables the lifting of movement restrictions on susceptible livestock during winter months during outbreaks, reducing the economic impact of bluetongue virus (BTV) which is a notifiable arbovirus of ruminants (Carpenter et al., 2009). Anecdotally, the seasonal period in the UK within which adult flight activity occurs has been lengthening for the past two decades (J. Boorman & P.S. Mellor, Personal Communication). This could not only reduce the utility of approaches that rely on reduced *Culicoides* adult activity, but also increase the probability of the arboviruses they transmit overwintering in the UK and becoming established, with significant consequences for livestock production.

Established in the 1960s, the Rothamsted Insect Survey network is the longest continuous survey of insects in the world (Harrington et al., 2007). The 12.2 m high suction‐traps collect a daily sample of aerial invertebrates that have been used to investigate changes in the population dynamics of pest aphid species (Harrington et al., 2007). Records of the first annual flight of pest aphid species show a significant change in the timing of first flight, with an average of 0.6 days per year earlier whilst last flight and abundance remained relatively constant (Bell et al., 2015).

The Rothamsted suction‐traps have been previously used to monitor *Culicoides* activity (Fassotte et al., 2008) and describe spatial and seasonal patterns of *Culicoides* flight in relation to meteorological conditions over a single season (Sanders et al., 2011). These collections provide a unique opportunity to examine the seasonal dynamics and abundance of adult vectors over the period of time relevant to that at which global climate has been changing. Here we use this resource to describe changes in the seasonality and abundance of *Culicoides* vectors of BTV in the Palearctic, at two sites over a period spanning nearly 40 years and investigate the role of local climate conditions and assess the likely impact of these changes on the transmission of arboviruses.

## 2 MATERIALS AND METHODS

### 2.1 Trap collections, climatic variables, and livestock density data

The Rothamsted suction‐trap network sites are situated in arable areas where *Culicoides* abundance is low and abundance in collections highly variable (Fassotte et al., 2008; Sanders et al., 2011). Two of the 12 UK sites, however, are situated in pastoral‐dominated habitat and had sufficient *Culicoides* abundance and temporal coverage to enable population changes over a period from 1974 to 2012 to be examined. The Preston, Lancashire (53°51’16”N, 2°45’48”W) and Starcross, Devon (50°37’44”N, 3°27’13”W) trap sites were selected in the north‐west and south‐west of England respectively, where different changes in climatic conditions have been experienced (Figure S1). The Preston trap is located within a diverse mixture of broad leaved woodland (27%), heathland (21%), and improved grassland (18%) with smaller proportions of sub‐urban (14%) and arable (10%) cover, whilst the area around Starcross is largely a mix of arable (66%) and suburban (20%) land cover with small amounts of improved grassland (10%).

Suction‐traps at both sites collected large numbers of *Culicoides* in 2008 (Sanders et al., 2011) and had near‐complete sample records from 1974 to 2012 from which *Culicoides* were identified. Samples were examined for *Culicoides* from every fourth day of every even year for Preston and every fourth day of every fourth year, with the addition of 1980, 2008, and 2012 for Starcross. The daily samples for both sites from 2008 (Sanders et al., 2011) were also included in the analysis.

*Culicoides* were counted and identified according to morphological keys (Campbell & Pelham‐Clinton, 1960; The Pirbright Institute, 2007) to species or species group level. Female *Culicoides* of the *Avaritia* subgenus were identified to the level of the polyphyletic Obsoletus group, described here as *Culicoides obsoletus* Meigen, *Culicoides scoticus* Downes and Kettle, *Culicoides dewulfi* Goetghebuer, and *Culicoides chiopterus* Meigen. The long‐term storage of these samples precluded molecular analysis to species level and separation of *C. dewulfi* and *C. chiopterus* by wing‐pattern morphology in older samples was considered to be unreliable. Males of the Obsoletus group were identified to species‐level and have been used previously as a proxy for the activity of the females of each species (Sanders et al., 2011; Searle et al., 2014). In addition, age grading from pigmentation of female abdomens was also precluded by the age and storage method of specimens.

The number of *Culicoides* present in large samples of more than 500 individual *Culicoides* was estimated using a randomised grid sampling method as described previously (Sanders et al., 2011). Eleven species/groups of *Culicoides* were recorded: total *Culicoides*, Obsoletus group females, *C. obsoletus s.s.* males, *C. scoticus* males, *C. chiopterus* males, *C. dewulfi* males, *C. pulicaris* L. females, *C. pulicaris* males, *C. punctatus* Meigen females, *C. punctatus* males, and other *Culicoides* (i.e., those species not listed).

Temperature and precipitation data for 1973–2012 were obtained from the UK Climate Projections (UKCP09) gridded observation data‐sets. These cover the UK at 5 km × 5 km resolution with the data for each trap site extracted for the grid square in which it is located (Figure S2a–d). Monthly soil moisture data for the trap sites were extracted from a 1 km resolution Grid to Grid hydrological model (Bell, Kay, Jones, & Moore, 2007) outputs (not observations) supplied by Centre for Ecology and Hydrology (Figure S2e). The North Atlantic Oscillation winter index, an indicator of winter weather severity, was taken from the Hurrell station‐based index, 1973–2012 (Figure S2f; Hurrell, 2014). In the absence of local livestock data over the sampling period, the numbers of cattle and sheep in each trapping area were taken from the corresponding 10 km by 10 km grid square extracted from the EDINA database of agricultural survey data 1972–2010 (Edina Agcensus, 2014) for census years (1976, 1979, 1981, 1988, 1993–1997, 2000, 2003–2004, and 2010) and a linear interpolation or extrapolation was used for non‐census years (Figure S3). Fine scale spatial information (25 m^2^ land parcels) on land cover during the latter half of the study period was derived from CEH Land Cover Maps (Appendix S1; Figure S4).

### 2.2 Statistical methods

Five measures of phenology and abundance of *Culicoides* biting midges were considered: (a) date of first appearance (defined as ≥ 5 individuals caught); (b) date of last appearance (defined as ≤ 5 individuals caught); (c) season length (i.e., time between first and last appearance); (d) maximum daily catch; and (e) mean daily catch (i.e., total number of midges caught divided by the number of trap days). The threshold number of individuals chosen for determining the dates of first and last appearance is based on the definition of the seasonal vector‐free period in European regulations on bluetongue (Commission Regulation (EC) No 1,266/2007, Annex V [European Commission, 2007]). However, preliminary exploratory analysis suggested that alternative thresholds, while changing the dates of first and last appearance (and, hence, season length), had no impact on the conclusions about trends in any of these phenology measures.

Annual trends in each of the measures were explored by computing them from the Rothamsted suction‐trap data, fitting a straight‐line relationship to the computed measure for each year by linear regression and testing whether the slope differed significantly (*p* < 0.05) from zero. Because data for all years (except one) are based on a subsample of the daily catches, the robustness of these trends was assessed by fitting a generalised linear model to the full Rothamsted suction‐trap (RST) dataset for the species/group and site in a Bayesian framework (cf. (Sanders et al., 2011)). In the model, the trap catch on day *t* was assumed to follow a Poisson distribution with mean *μ*(*t*) given by,

$$\left[\log\left(\mu \left(t\right)\right)=b_{0}+\sum_{n=1}^{2}\left\{a_{n}\sin\left(\frac{2n\pi }{365}t\right)+b_{n}\cos\left(\frac{2n\pi }{365}t\right)\right\},\right]$$

where *b*_0_ scales the abundance and the *a_n_*s and *b_n_*s are the seasonality parameters (i.e., they control phenology). The model parameters were allowed to vary between years (to allow for variation in seasonality and abundance between years) and the model also included temporal autocorrelation (to allow for dependence between observations) (see Appendix S2 for details). The fitted models were then used to generate replicated simulated phenology and abundance measures with the trends in these replicated measures analysed by linear regression (see Appendix S2 for details). In addition to assessing the robustness of the trends, this approach also allows a more detailed exploration of the underlying trends in the Rothamsted suction‐trap data and, in particular, to disentangle the roles of abundance and phenology from one another.

The relationships between the five phenology and abundance measures and climate were assessed for 19 summary climate variables and for density of two livestock species (Table S1). Following exploratory data analysis to assess the form of potential relationships, linear regression was used to fit a straight line relationship between each measure and the variable of interest and testing the significance of the slope. As with the annual trends, this was done using the measures computed from the data and using replicated simulated measures to assess the robustness of any relationships. Subsequently, multiple regression was carried out using a model including all those variables for which there was a significant univariable relationship with a measure. Model selection started from a model including all variables and proceeded by stepwise deletion of non‐significant (*p* > 0.05) terms. Again, this was done using the replicated simulated measures to assess the robustness of any relationships.

### 2.3 Implications for arbovirus transmission

To explore the possible consequences of changing *Culicoides* phenology on the transmission of *Culicoides*‐borne viruses we used a simple model for viral replication inside the adult midge vectors based on cumulative thermal time (Carpenter et al., 2011; Wilson, Carpenter, Gloster, & Mellor, 2007). In the model a *Culicoides* midge is able to transmit BTV once the accumulated degree‐days since it was infected equal or exceed the level required for completion of the extrinsic incubation period, with threshold temperature for viral replication and virus replication rate above threshold estimated from outbreaks of BTV‐8 in Great Britain (Sumner, Orton, Green, Kao, & Gubbins, 2017). Assuming no infected midges survive from one season to the next (Wilson, Darpel, & Mellor, 2008), we used the model to estimate the earliest date at which newly infected midges would first become infectious (because they have accumulated sufficient thermal time) and so the earliest time at which transmission could occur.

## 3 RESULTS

### 3.1 Culicoides samples

**Figure 1:**
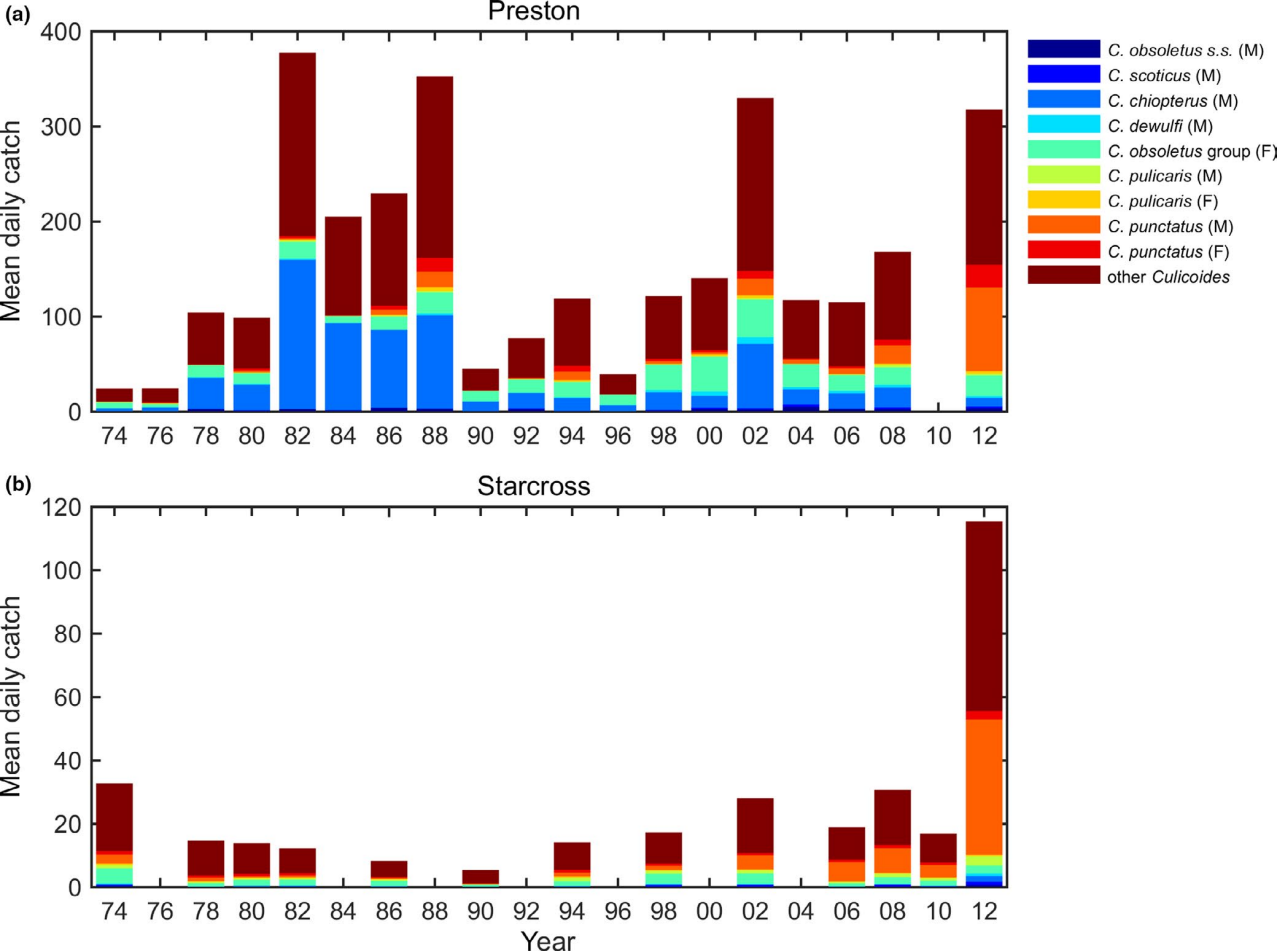
Mean daily catches of *Culicoides* biting midges in the Rothamsted Insect Survey suction traps at (a) Preston and (b) Starcross between 1974 and 2012. The colours indicate different *Culicoides* species/groups caught (see legend)

A total of 2,867 *Culicoides* samples from both sites were examined. At Preston, the 1,628 samples contained a total of 139,861 *Culicoides*; at Starcross the 1,239 samples contained 19,193 *Culicoides*. Samples at Preston were dominated by the Obsoletus group (65.0%), the Pulicaris group contributed 21.2%, with other species including *C. circumscriptus* Keiffer, *C. achrayi* Kettle and Lawson, *C. brunnicans* Edwards, *C. stigma* Meigen, *C. nubeculosus* Meigen, individually not exceeding 1% of the total catch and together accounting for 13.9% (Figure 1). At Starcross, the trap samples were dominated by the Pulicaris group (54.1%) and the Obsoletus group contributed 19.6%. Other species of *Culicoides*, including *C. circumscriptus* (3.8%), when combined contributed 26.2% to the total catch. At both sites the traps collected a greater proportion of male (68.4% at both sites) than female *Culicoides* (31.6%) (Figure 1).

### 3.2 Annual trends in phenology and abundance

The full results of the statistical modelling are presented in the supporting information, including assessment of the fit of the model to data (see Appendix S1; Figures S5–S26). Model checking indicated that the models adequately captured the data in terms of overall fit, total catch and maximum daily catch.

**Figure 2:**
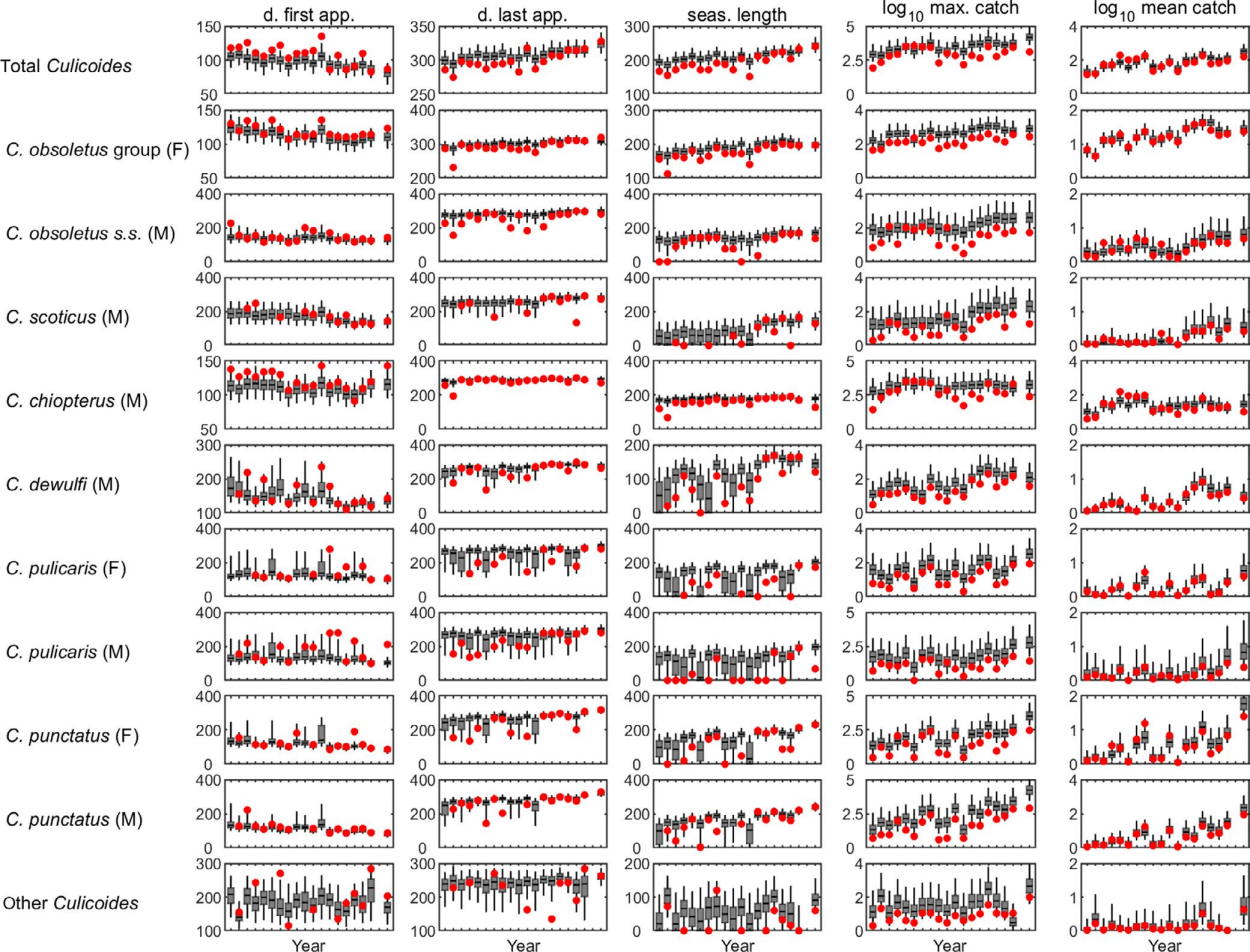
Annual trends in *Culicoides* phenology and abundance at Preston, 1974–2012. Results are presented for five measures: day (from 1 January) of first appearance (d. first app.); day (from 1 January) of last appearance (d. last app.); season length (seas. length; in days); log_10_ maximum daily catch (log_10_ max. catch); and log_10_ mean daily catch (log_10_ mean catch). In each figure the observed measures calculated from the trap catches are shown as red circles. The simulated measures generated from the generalised linear models fitted to the RST data are shown as box and whisker plots (median: horizontal black line; interquartile range: grey box; and 2.5th and 97.5th percentiles: whiskers)

**Figure 3:**
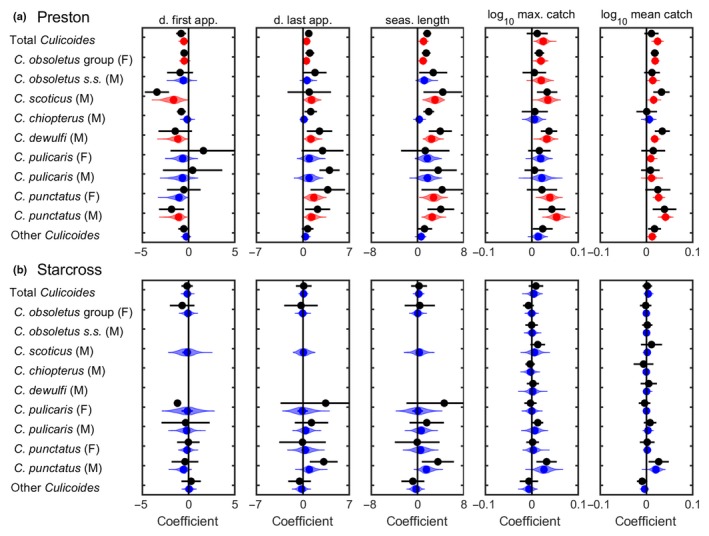
Summary of the annual trends in *Culicoides* phenology and abundance at (a) Preston and (b) Starcross, 1974–2012. Results are presented for five measures: day (from 1 January) of first appearance (d. first app.); day (from 1 January) of last appearance (d. last app.); season length (seas. length); log_10_ maximum daily catch (log_10_ max. catch); and log_10_ mean daily catch (log_10_ mean catch). Each figure shows the regression coefficients for the trend line for the measure estimated in two ways. First, the black circles and error bars show the estimate and 95% confidence interval, respectively, based on the measures computed directly from the Rothamsted Insect Survey suction trap data. Second, violin plots show the posterior density (shape), median (circle), and interquartile range (line) for the regression coefficient inferred from the posterior predictive distribution for the data. Plots are coloured red where evidence for the trend is robust (median posterior *p*‐value <0.05) and blue where it is not (median posterior *p*‐value >0.05). Where a plot is missing for Starcross, insufficient individuals were caught to be able to define the dates of first and last appearance

Between 1974 and 2012 at the Preston site, dates of first appearance were earlier, dates of last appearance were later, seasons were longer and abundance (maximum and mean daily catch) greater over time, though these trends were not significant for all 11 *Culicoides* species/groups (Table 1; Figure 2). By contrast, no significant trends were identified in any of the measures of phenology or abundance for any of the 11 species/groups at Starcross over the same time period (Table S2; Figure S27). These conclusions are robust to uncertainty in the phenology and abundance measures (Figure 3).

**Table 1:** Summary of annual trends in measures of *Culicoides* phenology and abundance at Preston, 1974–2012 (posterior median and 95% credible interval)

Species	Change (days per year)			% Change per year	
	Date of first appearance	Date of last appearance	Season length	Maximum daily catch	Mean daily catch
total *Culicoides*	**−0.5 (−0.8, −0.2)**	**0.5 (0.2, 0.9)**	**1.0 (0.6, 1.5)**	**5.8 (2.5, 9.1)**	**5.7 (3.9, 7.6)**
*C. obsoletus* group (♀)	**−0.5 (−0.8, −0.1)**	**0.5 (0.2, 0.8)**	**1.0 (0.5, 1.4)**	**4.6 (2.2, 7.1)**	**4.4 (3.3, 5.5)**
*C. obsoletus s.s.* (♂)	−0.6 (−1.4, 0.2)	0.6 (0.0, 1.4)	1.2 (0.1, 2.3)	**4.8 (0.9, 9.1)**	**3.2 (1.6, 5.2)**
*C. scoticus* (♂)	**−1.6 (−2.8, −0.5)**	**1.3 (0.4, 2.3)**	**3.0 (1.6, 4.3)**	**8.2 (3.7, 12.9)**	**3.6 (2.2, 5.4)**
*C. chiopterus* (♂)	−0.1 (−0.6, 0.3)	0.1 (−0.2, 0.5)	0.3 (−0.3, 0.9)	1.4 (−2.4, 4.9)	1.3 (−1.0, 3.4)
*C. dewulfi* (♂)	**−1.2 (−2.3, −0.4)**	**1.2 (0.4, 2.3)**	**2.4 (1.3, 3.7)**	**7.7 (4.4, 11.7)**	**4.1 (3.0, 5.5)**
*C. pulicaris* (♀)	−0.6 (−2.2, 0.5)	1.0 (−0.3, 2.7)	1.7 (−0.1, 3.5)	4.5 (0.7, 8.8)	**2.1 (0.9, 3.8)**
*C. pulicaris* (♂)	−0.7 (−2.2, 0.5)	0.9 (−0.4, 2.2)	1.7 (0.1, 3.5)	5.1 (0.2, 11.5)	**2.5 (0.4, 5.2)**
*C. punctatus* (♀)	−1.0 (−2.2, −0.4)	**1.6 (0.5, 2.9)**	**2.8 (1.2, 4.5)**	**9.3 (4.8, 13.6)**	**6.2 (4.4, 8.4)**
*C. punctatus* (♂)	**−1.1 (−2.4, −0.5)**	**1.3 (0.5, 2.6)**	**2.5 (1.3, 4.0)**	**13.0 (8.3, 17.7)**	**9.9 (7.6, 12.7)**
other *Culicoides*	−0.3 (−0.6, 0.1)	0.4 (−0.1, 0.8)	0.6 (0.0, 1.2)	3.3 (0.4, 6.5)	**2.9 (1.4, 4.3)**

Note

For those shown in bold, the median posterior‐predictive *p*‐value <0.05.

At Preston, the date of first appearance was 0.5–1.5 days per year earlier for total *Culicoides*, *C. obsoletus* group females, *C. scoticus* males, *C. dewulfi* males, and *C. punctatus* males (Table 1). Furthermore, the date of last appearance was delayed by a similar amount for these species/groups (Table 1). Accordingly, the length of the active season for these species/groups increased by around 1–3 days per year (Table 1). In addition, the date of last appearance was delayed by around two days per year and season length increased by about three days per year for *C. punctatus* females (Table 1). There were increases in abundance of between 5% and 13% per year for seven (out of 11) species/groups as measured by maximum daily catch and of between 2% and 10% per year for ten (out of 11) species/groups as measured by mean daily catch (Table 1).

In the underlying statistical models used to infer the annual trends in phenology and abundance measures, there were no significant trends in any of the seasonality parameters (i.e., the *a_n_*s and *b_n_*s) for any of the species at either Preston or Starcross, nor were there significant trends in the abundance parameter (i.e., *b*_0_) for any of the species at Starcross (Figure S28). By contrast, the abundance parameters increased significantly over time for nine (out of 11) species at Preston (Figure S28) indicating that the earlier dates of first appearance and later dates of last appearance (Table 1) are a result of increased abundance (and, hence, an increased chance of being trapped) rather than to changes in phenology.

### 3.3 Climate, host, and land use variables

There was a significant increase in annual mean temperature at Preston (*b* = 0.03; *p* < 0.001) between 1974 and 2012 (Figure S2a), but no significant trends in either annual total precipitation (*b* = 2.1; *p* = 0.13) (Figure S2b) or annual mean soil moisture (*b* = 0.16, *p* = 0.58) (Figure S2e). There was a significant decrease in cattle density (*b *= −0.05, *p* = 0.05) and a significant increase in sheep density (*b* = 0.44, *p* = 0.01) between 1974 and 2012 at the site (Figure S4). At Starcross, there was a significant increase in annual mean temperature (*b* = 0.02; *p* < 0.001) over the same time period (Figure S2a), but no significant trends in annual total precipitation (*b* = 1.5, *p* = 0.19) (Figure S2b) or in annual mean soil moisture (*b* = 0.36, *p* = 0.13) (Figure S2e). Over the same time period there was a significant decrease in cattle density (*b*=−0.10, *p* < 0.001), but no significant trend in sheep density (*b* = 0.02, *p* = 0.64) (Figure S3).

### 3.4 Variables driving the trends in phenology and abundance measures

**Figure 4:**
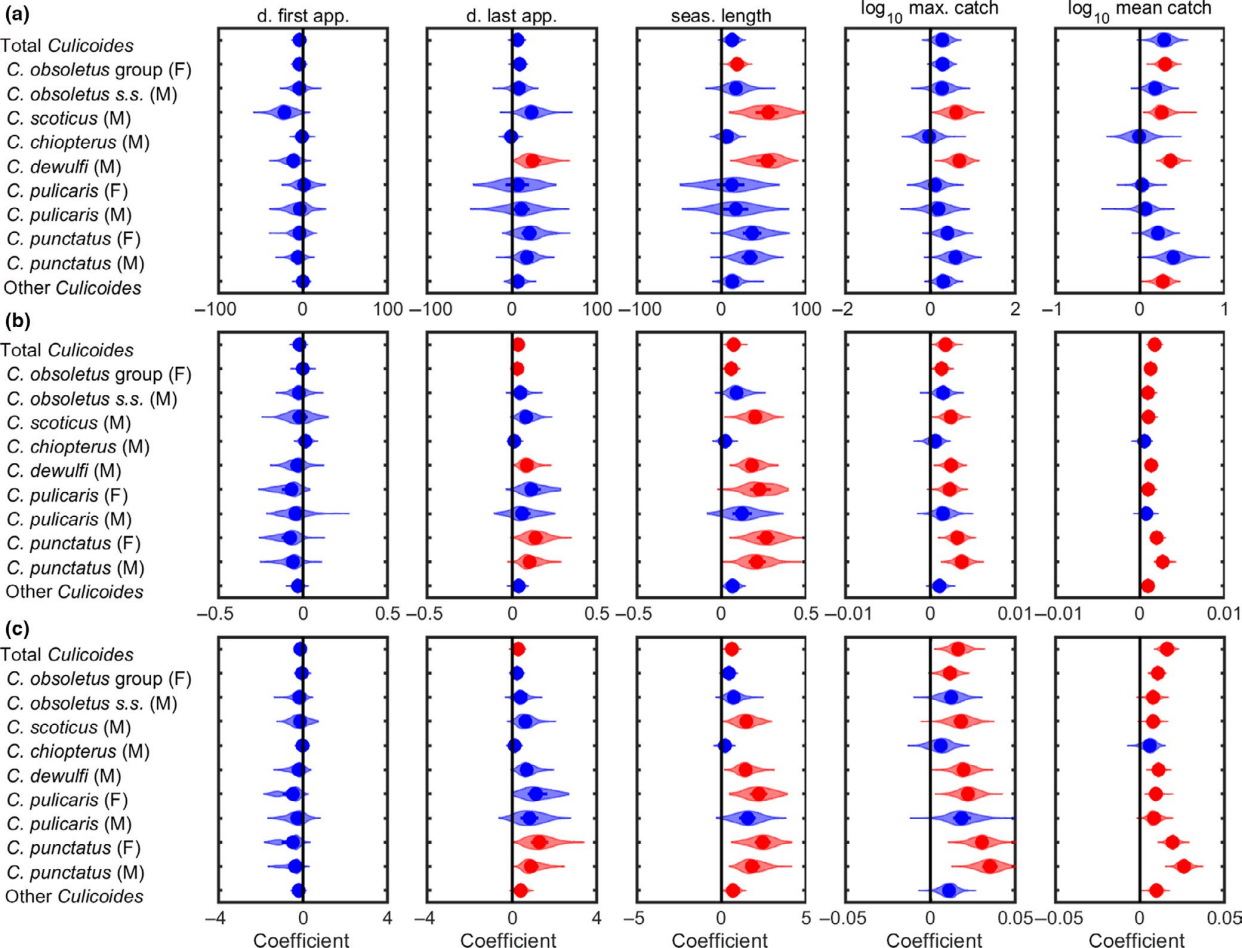
Relationship between (a) annual mean temperature, (b) annual total precipitation, and (c) annual mean soil moisture and measures of *Culicoides* phenology and abundance at Preston, 1974–2012. Results are presented for five measures: day (from 1 January) of first appearance (d. first app.); day (from 1 January) of last appearance (d. last app.); season length (seas. length); log_10_ maximum daily catch (log_10_ max. catch); and log_10_ mean daily catch (log_10_ mean catch). Each figure shows the posterior predictive distribution for the regression coefficient in a straight‐line relationship between the climate variable and the measure. Violin plots show the posterior density (shape), median (circle), and interquartile range (line) for the coefficient. Plots are coloured red where evidence for the trend is robust (median posterior *p*‐value <0.05) and blue where it is not (median posterior *p*‐value >0.05)

At Preston, higher annual mean temperatures, annual total precipitation, and annual mean soil moisture were associated with later dates of last appearance and longer seasons, as well as with higher maximum daily catches and mean daily catches (Figure 4). Furthermore, these associations were significant and consistent across a number of *Culicoides* species/groups (Figure 4). At Starcross, there were far fewer significant relationships than at Preston. Annual total precipitation was associated with an increase in mean catch and annual mean soil moisture was associated with an increase in season length, maximum catch, or mean catch for a small number (≤4) of species/groups (Figure S29). For the remaining climate variables (Table S1) at Preston and Starcross there was little evidence for any significant relationships with the abundance or phenology measures. None of the phenology and abundance measures were significantly associated with cattle or sheep densities at Preston or Starcross.

The multiple regression analysis was carried out for Preston only and included annual mean temperature, annual total precipitation, and annual mean soil moisture. The results suggest that there is limited evidence for multiple climate drivers acting together, because most final models include only a single climate variable (Figure S30). The only exception was for *C. dewulfi* males, where the maximum and mean daily catches were influenced by both annual mean temperature and annual mean soil moisture in combination (Figure S30).

### 3.5 Implications for transmission of *Culicoides*‐borne viruses

**Figure 5:**
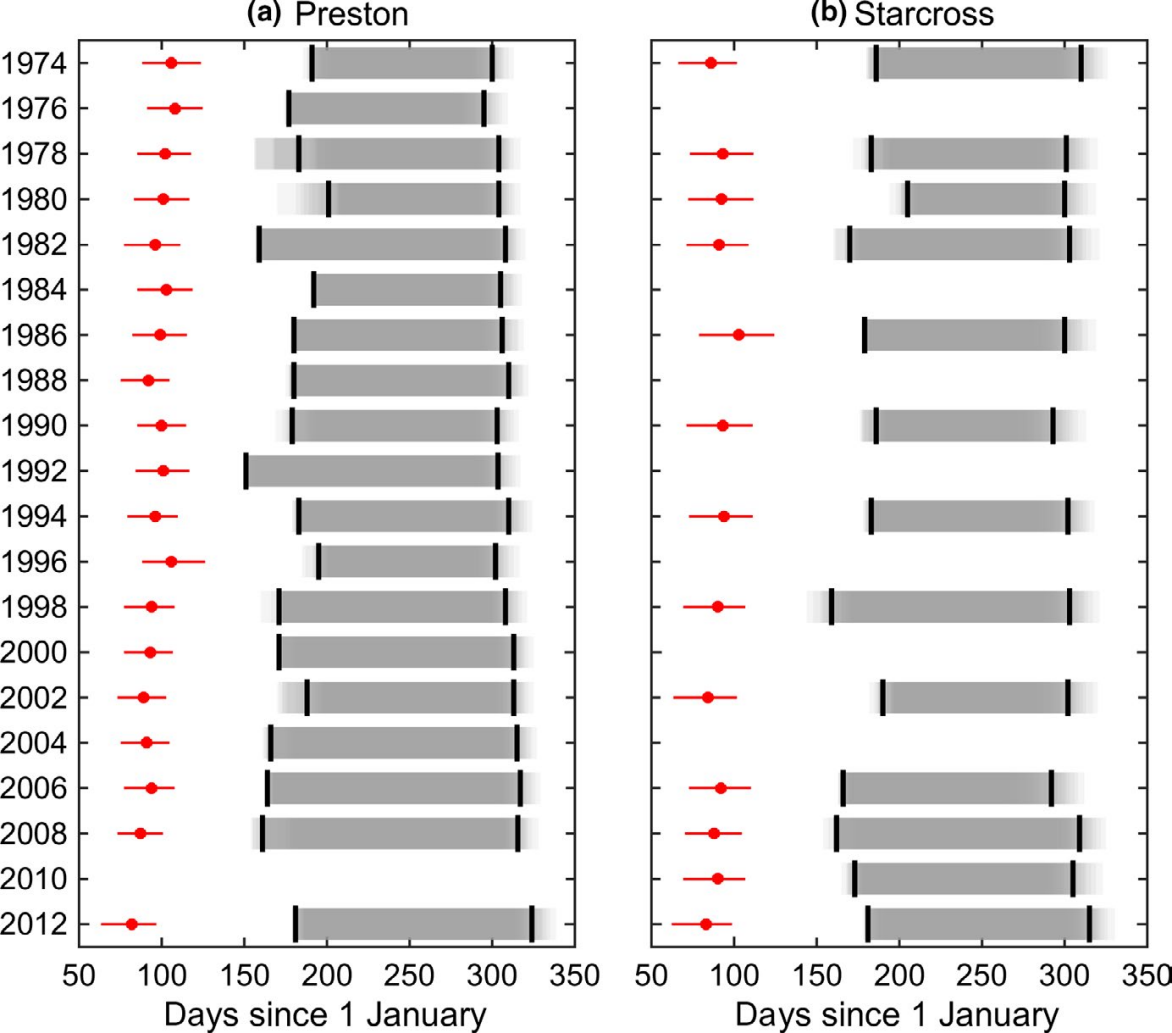
Transmission season for bluetongue virus at (a) Preston and (b) Starcross, 1974–2012. Each plot shows the date of first appearance (posterior median and 95% credible interval in red) and the estimated transmission season based on the earliest date at which a newly infected midge could become infectious and the date of last appearance (black lines show the posterior medians and grey shading shows five percentile bands from the fifth to the 95th percentile). Results are based on the total number of *Culicoides* biting midges caught

Using the model for viral replication there was no significant annual trend in the date at which newly infected *Culicoides* would become infectious at either Preston (*b *= −0.36, *p* = 0.16) or Starcross (*b *= −0.32, *p* = 0.16). Comparison of the date at which newly infected *Culicoides* would become infectious, with the dates of first appearance shows that midges are active for several months before virus transmission could occur (Figure 5). Consequently, earlier first appearance is unlikely to influence the spread of BTV in the UK. Once midges become infectious, they remain so for the rest of their life. Consequently, the transmission season will end only once adult vectors cease to be active and complete gonadotrophic cycles. Accordingly, a later date of last appearance results in a longer transmission season (Figure 5).

## 4 DISCUSSION

This study demonstrates that significant changes in the first and last date of appearance of adult *Culicoides* in the UK have occurred in response to climate drivers over 40 years, but that these changes are heterogeneous across sites. While conducted at a single‐country scale and at a logistically restricted number of sites, this is the first time that changes in adult phenology have been directly related to climate parameters at this time scale for this vector group and for vectors of medical and veterinary pathogens as a whole. Previous studies have either lacked the standardisation of trapping method (through replacement with new designs over time as they became available), or have been conducted in parallel with attempts at control (e.g., long term monitoring of mosquito populations in Singapore (Ooi, Goh, & Gubler, 2006)).

The increase in the length of the adult flight activity season was related to a significant increase over time in *Culicoides* abundance (driven by temperature, precipitation, and soil moisture), that could not be explained by other changes in the environment and would be likely to increase the probability of arbovirus transmission and overwintering at the site. The study also emphasises heterogeneity in response that will be challenging to capture as part of applied attempts to predict future risks of arbovirus transmission and overwintering. At −0.5–−1.5 days per year, the observed rate of advancement in the first capture of adult *Culicoides* at Preston are greater than the reported average for phenological events of terrestrial invertebrates of −0.4 days per year (Thackeray et al., 2016, 2010) and similar to that observed in some Lepidoptera and Homoptera (Bell et al., 2015; Roy & Sparks, 2000). The putative vector species of BTV in the northern Palaearctic, with the exception of *C. chiopterus*, demonstrated significant changes in date of first appearance, last appearance, or increases in mean catch at the Preston site. At Starcross significant changes in precipitation were not observed, and no significant trends in *Culicoides* abundance or phenology were observed. Differences in sampling intensity, *Culicoides* abundance and fauna between the sites were not responsible for the difference in changes observed.

Attributing changes in phenology to climate change is problematic due to the plasticity in response of species to short‐term changes in factors independent of meteorological conditions (Diez et al., 2012). Mean temperature and precipitation were the only significant explanatory variables associated with the increase in activity and changes in phenology seen at Preston. Despite the geographical separation of Preston and Starcross (435 km), a similar change in average temperature occurred at both sites. The significant increase in precipitation observed at Preston was not observed at Starcross. The major land use changes seen at both trap sites are likely to have a neutral or negative impact on breeding site and host availability for *Culicoides* (Purse et al., 2015, 2005). Although livestock density, in particular that of sheep and cattle, is a key driver of vector *Culicoides* abundance in providing hosts and associated breeding habitats (Searle et al., 2014), changes in host livestock density were not associated with the changes observed.

Soil type and soil moisture determine *Culicoides* larval habitat suitability and availability (Mellor, Boorman, & Baylis, 2000; Purse et al., 2015). The sandy soil at Starcross retains lower soil moisture than the clay loam at Preston. Ephemeral larval habitats may dry more quickly and therefore habitat suitability and availability dependent on precipitation rather than temperature may be limiting the *Culicoides* population at the site. There were sufficient *Culicoides* data from Starcross for our analysis to identify similar degrees of change to that observed at Preston. The species complement at each site was not responsible for the difference in observed changes: significant shifts trends in the Pulicaris group species collection at Preston were not observed at Starcross where the species group dominated samples.

Analysis of the underlying statistical models that were used to examine the data suggests that the observed changes in phenology of *Culicoides* at Preston are a result of increased abundance of *Culicoides*. Increased abundance increases the likelihood of collection, extending the period when the number of actively flying adults is great enough for them to appear in samples. No evidence for changes in the timing or pattern of peaks in activity of *Culicoides*, or impacts of previous year's meteorological conditions was found. The increase in abundance of *Culicoides* at Preston was driven by the impact of warmer temperatures and increased precipitation experienced at the site over time. The RSTs collect *Culicoides* undergoing active dispersal flight at 12.2 m, with a male bias and lower trapping efficiency than the standard UV light‐suction traps (Sanders et al., 2011). In the absence of comparative studies, there is no evidence that these collections are not proportional to the population engaged in host‐seeking behaviour closer to the ground.

The extension of the active period of approximately 40 days over the study period has significant implications for the transmission of viruses by *Culicoides*. The transmission of *Culicoides*‐borne pathogens can only occur during periods of adult vector activity. The emergence of *Culicoides* earlier in the spring may not lead to increased or earlier transmission as activity occurs below the replication threshold temperature of the virus. The increased activity season of *Culicoides* observed at Preston would allow additional cycles of transmission at the end of the season as infected *Culicoides* would be active for longer. In temperate areas, the “seasonal vector‐free period” is used to delineate periods of low risk of transmission to allow livestock movement across protection zones of different disease status (Wilson & Mellor, 2009). At Preston, this period has effectively reduced by 40 days over the period 1974–2012, reducing the time over which a virus such as BTV would have had to survive in vertebrate hosts. Warming winter temperatures may also promote survival of infected *Culicoides* adults and potential transmission of viruses in microclimates such as large indoor animal holdings (Sarvasova, Kocisova, Liptakova, Hlavata, & Mathieu, 2016). Extended adult *Culicoides* activity into autumn was predicted to increase the impact and occurrence of clinical signs of Schmallenberg virus and the likelihood of transmission of BTV by endophilic *Culicoides* in winter (Bessell et al., 2013; Napp et al., 2011; Searle et al., 2014).

This study is the first to examine long‐term changes in the phenology and abundance of arbovirus vectors, using a unique data set of standardised trapping over 40 years with observed climatic changes. The study demonstrates trends in abundance and phenology of *Culicoides* vectors that vary between species and sites are sensitive to local variation in climate change. Rather than use a simple correlation with climate changes we examined other changes in the potential drivers of vector population dynamics, host density and land use, qualifying that the changes observed are likely to be due to the observed changes in climate. It is essential to understand the response of vector populations to climate change within the context of change in other ecological drivers. Large scale projections of the response of a vector group to climate change using a functional relationship between vector biology and climate are unable to fully account for the variability both in local population response and the local effects of climate change. The impact of future changes in climate on the population dynamics of arbovirus vectors such as *Culicoides* will be regulated by local biotic and abiotic factors that govern the capacity of a site to support the population.

## AUTHORS’ CONTRIBUTIONS

C.J.S. wrote the manuscript and with C.R.S. was responsible for development of the study and insect identification with the aid of M.E. L.B. provided advice on meteorological and climate data, B.P. provided advice on impacts of climate, land use and hydrology, R.H. and S.C. oversaw study design and direction and S.G. undertook the modelling of climate variables and *Culicoides* seasonality. All authors contributed to the final version of the manuscript.

## Data Availability

Data available from the Dryad Digital Repository https://doi.org/10.5061/dryad.h7b12g4 (Sanders et al., 2019).

## References

[jpe13415-bib-0001] Altizer, S. , Dobson, A. , Hosseini, P. , Hudson, P. , Pascual, M. , & Rohani, P. (2006). Seasonality and the dynamics of infectious diseases. Ecology Letters, 9, 467–484. 10.1111/j.1461-0248.2005.00879.x 16623732

[jpe13415-bib-0002] Bell, J. R. , Alderson, L. , Izera, D. , Kruger, T. , Parker, S. , Pickup, J. , … Harrington, R. (2015). Long‐term phenological trends, species accumulation rates, aphid traits and climate: Five decades of change in migrating aphids. Journal of Animal Ecology, 84, 21–34. 10.1111/1365-2656.12282 25123260 PMC4303923

[jpe13415-bib-0003] Bell, V. , Kay, A. L. , Jones, R. G. , & Moore, R. J. (2007). Development of a high resolution grid‐based river flow model for use with regional climate model output. Hydrology and Earth System Sciences, 11, 532–549. 10.5194/hess-11-532-2007

[jpe13415-bib-0004] Bessell, P. R. , Searle, K. R. , Auty, H. K. , Handel, I. G. , Purse, B. V. , & Bronsvoort, B. M. D. (2013). Epidemic potential of an emerging vector borne disease in a marginal environment: Schmallenberg in Scotland. Scientific Reports, 3 10.1038/srep01178 PMC3560360 23378911

[jpe13415-bib-0005] Campbell, J. A. , & Pelham‐Clinton, E. C. . (1960). Taxonomic review of the British species of *Culicoides* Latreille (Diptera, Ceratopogonidae). Proceedings of the Royal Entomological Society of London (B), 67, 181–302.

[jpe13415-bib-0006] Carpenter, S. , Wilson, A. , Barber, J. , Veronesi, E. , Mellor, P. , Venter, G. , & Gubbins, S. (2011). Temperature dependence of the extrinsic incubation period of Orbiviruses in *Culicoides* biting midges. PLoS ONE, 6, e27987. 22125649 10.1371/journal.pone.0027987 PMC3220716

[jpe13415-bib-0007] Carpenter, S. , Wilson, A. , & Mellor, P. S. (2009). *Culicoides* and the emergence of bluetongue virus in northern Europe. Trends in Microbiology, 17, 172–178. 10.1016/j.tim.2009.01.001 19299131

[jpe13415-bib-0008] Cleland, E. E. , Chuine, I. , Menzel, A. , Mooney, H. A. , & Schwartz, M. D. (2007). Shifting plant phenology in response to global change. Trends in Ecology & Evolution, 22, 357–365. 10.1016/j.tree.2007.04.003 17478009

[jpe13415-bib-0009] Diez, J. M. , Ibanez, I. , Miller‐Rushing, A. J. , Mazer, S. J. , Crimmins, T. M. , Crimmins, M. A. , … Inouye, D. W. (2012). Forecasting phenology: From species variability to community patterns. Ecology Letters, 15, 545–553. 10.1111/j.1461-0248.2012.01765.x 22433120

[jpe13415-bib-0010] Edina Agcensus . (2014). Agricultural census. Retrieved from https://access.edina.ac.uk/agcensus/

[jpe13415-bib-0011] Elbers, A. R. W. , Koenraadt, C. J. M. , & Meiswinkel, R. (2015). Mosquitoes and *Culicoides* biting midges: Vector range and the influence of climate change. Revue Scientifique Et Technique‐Office International Des Epizooties, 34, 123–137. 10.20506/rst.34.1.2349 26470453

[jpe13415-bib-0012] European Commission . (2007). European Commission Regulation (EC) No 1266/2007 of 26 October 2007 on implementing rules for Council Directive 2000/75/EC as regards the control, monitoring, surveillance and restrictions on movements of certain animals of susceptible species in relation to bluetongue.

[jpe13415-bib-0013] Fassotte, C. , Delecolle, J. C. , Cors, R. , Defrance, T. , De Deken, R. , Haubruge, E. , & Losson, B. (2008). *Culicoides* trapping with Rothamsted suction traps before and during the bluetongue epidemic of 2006 in Belgium. Preventive Veterinary Medicine, 87, 74–83. 10.1016/j.prevetmed.2008.06.007 18640735

[jpe13415-bib-0014] Forrest, J. , & Miller‐Rushing, A. J. (2010). Toward a synthetic understanding of the role of phenology in ecology and evolution. Philosophical Transactions of the Royal Society B‐Biological Sciences, 365, 3101–3112. 10.1098/rstb.2010.0145 PMC2981948 20819806

[jpe13415-bib-0015] Gould, E. A. , & Higgs, S. (2009). Impact of climate change and other factors on emerging arbovirus diseases. Transactions of the Royal Society of Tropical Medicine and Hygiene, 103, 109–121. 10.1016/j.trstmh.2008.07.025 18799177 PMC2915563

[jpe13415-bib-0016] Gould, E. A. , Pettersson, J. , Higgs, S. , Charrel, R. , & de Lamballerie, X. (2017). Emerging arboviruses: Why today? One Health, 4, 1–13. 10.1016/j.onehlt.2017.06.001 28785601 PMC5501887

[jpe13415-bib-0017] Gubbins, S. , Carpenter, S. , Baylis, M. , Wood, J. L. N. , & Mellor, P. S. (2008). Assessing the risk of bluetongue to UK livestock: Uncertainty and sensitivity analyses of a temperature‐dependent model for the basic reproduction number. Journal of the Royal Society Interface, 5, 363–371. 10.1098/rsif.2007.1110 PMC2497440 17638649

[jpe13415-bib-0018] Harrington, R. , Clark, S. J. , Welham, S. J. , Verrier, P. J. , Denholm, C. H. , Hulle, M. , … European Union Examine Consortium . (2007). Environmental change and the phenology of European aphids. Global Change Biology, 13, 1550–1564. 10.1111/j.1365-2486.2007.01394.x

[jpe13415-bib-0019] Hurrell, J. (2014). The climate data guide: Hurrell North Atlantic Oscillation (NAO) index (station based). eds National Center for Atmospheric Research Staff. Retrieved from https://climatedataguide.ucar.edu/climate-data/hurrell-north-atlantic-oscillation-nao-index-station-based

[jpe13415-bib-0020] Kovats, R. S. , Campbell‐Lendrum, D. H. , McMichael, A. J. , Woodward, A. , & Cox, J. S. (2001). Early effects of climate change: Do they include changes in vector‐borne disease? Philosophical Transactions of the Royal Society of London Series B‐Biological Sciences, 356, 1057–1068. 10.1098/rstb2001.0894 PMC1088500 11516383

[jpe13415-bib-0021] Lafferty, K. D. (2009). The ecology of climate change and infectious diseases. Ecology, 90, 888–900. 19449681 10.1890/08-0079.1

[jpe13415-bib-0022] Liang, G. , Gao, X. , & Gould, E. A. (2015). Factors responsible for the emergence of arboviruses; strategies, challenges and limitations for their control. Emerging Microbes and Infections, 4, e18 10.1038/emi.2015.1018 26038768 PMC4395659

[jpe13415-bib-0023] MacLachlan, N. J. , & Guthrie, A. J. (2010). Re‐emergence of bluetongue, African horse sickness, and other Orbivirus diseases. Veterinary Research, 41, 35 10.1051/vetres/2010007 20167199 PMC2826768

[jpe13415-bib-0024] Mellor, P. S. , Boorman, J. , & Baylis, M. (2000). *Culicoides* biting midges: Their role as arbovirus vectors. Annual Review of Entomology, 45, 307–340. 10.1146/annurev.ento.45.1.307 10761580

[jpe13415-bib-0025] Menzel, A. , Sparks, T. H. , Estrella, N. , Koch, E. , Aasa, A. , Ahas, R. , … Zust, A. (2006). European phenological response to climate change matches the warming pattern. Global Change Biology, 12, 1969–1976.

[jpe13415-bib-0026] Napp, S. , Gubbins, S. , Calistri, P. , Allepuz, A. , Alba, A. , Garcia‐Bocanegra, I. , … Casal, J. (2011). Quantitative assessment of the probability of bluetongue virus overwintering by horizontal transmission: Application to Germany. Veterinary Research, 42, 4 10.1186/1297-9716-1142-1184 21314966 PMC3031226

[jpe13415-bib-0027] Ooi, E. E. , Goh, K. T. , & Gubler, D. J. (2006). Dengue prevention and 35 years of vector control in Singapore. Emerging Infectious Diseases, 12, 887–893. 10.3201/10.3201/eid1206.051210 16707042 PMC3373041

[jpe13415-bib-0028] Purse, B. V. , Carpenter, S. , Venter, G. J. , Bellis, G. , & Mullens, B. A. (2015). Bionomics of temperate and tropical *Culicoides* midges: Knowledge gaps and consequences for transmission of *Culicoides*‐borne viruses. Annual Review of Entomology, 60, 373–392. 10.1146/annurev-ento-010814-020614 25386725

[jpe13415-bib-0029] Purse, B. V. , Mellor, P. S. , Rogers, D. J. , Samuel, A. R. , Mertens, P. P. C. , & Baylis, M. (2005). Climate change and the recent emergence of bluetongue in Europe. Nature Reviews Microbiology, 3, 171–181. 10.1038/nrmicro1090 15685226

[jpe13415-bib-0030] Rafferty, N. E. , CaraDonna, P. J. , Burkle, L. A. , Iler, A. M. , & Bronstein, J. L. (2013). Phenological overlap of interacting species in a changing climate: An assessment of available approaches. Ecology and Evolution, 3, 3183–3193. 24102003 10.1002/ece3.668 PMC3790560

[jpe13415-bib-0031] Rogers, D. J. , & Randolph, S. E. ( 2006). Climate change and vector‐borne diseases In Hay S. I., Graham A. & Rogers D. J. (Eds.), Advances in parasitology: Global mapping of infectious diseases: methods, examples and emerging applications(Vol. 62, pp. 345–381). London, UK: Academic Press. 10.1016/S0065-308X(05)62010-6 16647975

[jpe13415-bib-0032] Root, T. L. , Price, J. T. , Hall, K. R. , Schneider, S. H. , Rosenzweig, C. , & Pounds, J. A. (2003). Fingerprints of global warming on wild animals and plants. Nature, 421, 57–60. 12511952 10.1038/nature01333

[jpe13415-bib-0033] Roy, D. B. , & Sparks, T. H. (2000). Phenology of British butterflies and climate change. Global Change Biology, 6, 407–416. 10.1046/j.1365-2486.2000.00322.x

[jpe13415-bib-0034] Sanders, C. J. , Shortall, C. R. , England, M. E. , Harrington, R. , Purse, B. V. , Burgin, L. , … Gubbins, S. (2019). Data from: Long‐term shifts in the seasonal abundance of adult *Culicoides* biting midges and their impact on the potential for arbovirus outbreaks. Dryad Digital Repository. 10.5061/dryad.h7b12g4 PMC6618056 31341330

[jpe13415-bib-0035] Sanders, C. J. , Shortall, C. R. , Gubbins, S. , Burgin, L. , Gloster, J. , Harrington, R. , … Carpenter, S. (2011). Influence of season and meteorological parameters on flight activity of *Culicoides* biting midges. Journal of Applied Ecology, 48, 1355–1364. 10.1111/j.1365-2664.2011.02051.x

[jpe13415-bib-0036] Sarvasova, A. , Kocisova, A. , Liptakova, E. , Hlavata, H. , & Mathieu, B. (2016). First insights into indoor and outdoor *Culicoides* activity related to the risk period for Bluetongue virus transmission in Eastern Slovakia. Acta Parasitologica, 61, 743–755. 27787226 10.1515/ap-2016-0103

[jpe13415-bib-0037] Searle, K. R. , Barber, J. , Stubbins, F. , Labuschagne, K. , Carpenter, S. , Butler, A. , … Purse, B. V. (2014). Environmental drivers of *Culicoides* phenology: How important is species‐specific variation when determining disease policy? PLoS ONE, 9, e111876. 25386940 10.1371/journal.pone.0111876 PMC4227682

[jpe13415-bib-0038] Sumner, T. , Orton, R. J. , Green, D. M. , Kao, R. R. , & Gubbins, S. (2017). Quantifying the roles of host movement and vector dispersal in the transmission of vector‐borne diseases of livestock. Plos Computational Biology, 13 10.1371/journal.pcbi.1005470 PMC5393902 28369082

[jpe13415-bib-0039] Tabachnick, W. J. (2016). Climate change and the arboviruses: Lessons from the evolution of the dengue and yellow fever viruses. Annual Review of Virology, 3, 125–145. 10.1146/annurev-virology-110615-035630 27482902

[jpe13415-bib-0040] Thackeray, S. J. , Henrys, P. A. , Hemming, D. , Bell, J. R. , Botham, M. S. , Burthe, S. , … Wanless, S. (2016). Phenological sensitivity to climate across taxa and trophic levels. Nature, 535, 241–U294. 27362222 10.1038/nature18608

[jpe13415-bib-0041] Thackeray, S. J. , Sparks, T. H. , Frederiksen, M. , Burthe, S. , Bacon, P. J. , Bell, J. R. , … Wanless, S. (2010). Trophic level asynchrony in rates of phenological change for marine, freshwater and terrestrial environments. Global Change Biology, 16, 3304–3313.

[jpe13415-bib-0042] The Pirbright Institute . (2007). Pictorial guide to the wings of British *Culicodies* (Diptera: Ceratopogonidae). Retrieved February 5, 2012, from www.Culicoides.net

[jpe13415-bib-0043] Weaver, S. C. , & Reisen, W. K. (2010). Present and future arboviral threats. Antiviral Research, 85, 328–345. 19857523 10.1016/j.antiviral.2009.10.008 PMC2815176

[jpe13415-bib-0044] Wilson, A. , Carpenter, S. , Gloster, J. , & Mellor, P. (2007). Re‐emergence of bluetongue in northern Europe in 2007. Veterinary Record, 161, 487–489. 17921441 10.1136/vr.161.14.487

[jpe13415-bib-0045] Wilson, A. , Darpel, K. , & Mellor, P. S. (2008). Where does bluetongue virus sleep in the winter? Plos Biology, 6, 1612–1617. 10.1371/journal.pbio.0060210 PMC2525685 18752350

[jpe13415-bib-0046] Wilson, A. , & Mellor, P. (2009). Bluetongue in Europe: Past, present and future. Philosophical Transactions of the Royal Society B‐Biological Sciences, 364, 2669–2681. 10.1098/rstb.2009.0091 PMC2865089 19687037

