# Supplementary material for: Long‐term shifts in the seasonal abundance of adult Culicoides biting midges and their impact on potential arbovirus outbreaks
Source: J Appl Ecol. 2019 May 29;56(7):1649–60. doi: 10.1111/1365-2664.13415 (PMC6618056; doi:10.1111/1365-2664.13415)
Supplement: Supplementary file 1 [file JPE-56-1649-s001.pdf]

Supporting information

Long-term shifts in the seasonal abundance of adult *Culicoides* biting midges and their impact on the potential for arbovirus outbreaks

Christopher J. Sanders[1], Chris R. Shortall[2], Marion England[1], Richard Harrington[2], Beth Purse[3], Laura Burgin[4], Simon Carpenter[1] and Simon Gubbins[1]

1. The Pirbright Institute, Pirbright, UK GU24 0NF
2. Rothamsted Research, Harpenden, UK AL5 2JQ
3. Centre for Ecology and Hydrology, Wallingford, UK OX10 8BB
4. The Met Office, Exeter, UK EX1 3PB

Appendix S1 Recorded land cover changes in Preston and Starcross suction trap sites over the study period

Fine scale spatial information ($25m^2$ land parcels) on land cover during the latter half of the study period was derived from the CEH Land Cover Maps for 1990 (Fuller *et al*. 1994), 2000 (Fuller *et al*. 2002) and 2007 (Morton *et al*. 2011). Though remote sensing methodologies differ between these Land Cover Map versions, the aggregate, coarse classes of habitat are broadly comparable. There are ten aggregate types but the coastal aggregate type was excluded since the habitats within this type such as littoral and sub-littoral rock and sediment should remain static between periods. Of these aggregated classes, broad-leaved woodland, semi-natural grassland and heathland are hypothesised to be the classes most likely to support hosts and breeding sites for *Culicoides*. To account for variability in methodology and possibility of mis-classification between periods, only losses or gains in cover exceeding 10% were considered as substantial.

Land cover and land cover change around trap sites was characterised as percentage cover and change in percentage cover of land within buffer zones surrounding the trap sites. Land cover was derived from CEH land cover maps (see supporting information). In the immediate vicinity (250m) of the Preston trap is a diverse mixture of broad leaved woodland (27%), heathland (21%) and improved grassland (18%) with smaller proportions of sub-urban (14%) and arable (10%) cover. Within 2km of the trap site the landscape is dominated by improved grassland (65%) and arable land (19%). The area around Starcross is largely a mix of arable (66%) and suburban (20%) land cover with small amounts of improved grassland (10%). Within 2 km of the trap site, arable (34%) and coastal (28%) dominate with some broadleaved woodland (11%) and improved grassland (11%). A buffer zone of 250m was selected to reflect cover and changes within the immediate vicinity of trap sites whilst a buffer zone of 2km was selected to be equivalent of the larger spatial scale over which these traps may "attract" insects. Since 1990, there has been a large decrease in the proportion of semi-natural grassland (-23%) within a 2km radius of the Preston site, with an increase in improved grassland (+15%) and small increases in arable and broad leaf woodland (1% and 2% respectively) (Figure S3). Since 1990, the area within a 2km radius of the Starcross trap there has been a decline in semi-natural grassland (-10%), and an increase in broadleaf woodland (8%) and arable land (6%). Soil type differs between the sites, with Starcross having a sandy soil that does not retain as much moisture as the clay loam at Preston.

Appendix S2 Bayesian methods for inferring trends in phenology and abundance

S2.1 Modelling approach

The Rothamsted Insect Survey suction trap (RST) data were analysed using generalised linear models assuming Poisson errors and a log link function (cf. Sanders *et al.* 2011). The models included seasonality, temporal autocorrelation amongst the observations (to allow for dependence between observations) and hierarchical structure in the model parameters (to allow for inter-annual differences). Separate models were developed for each of the eleven species/group at Preston and Starcross (i.e. a total of 22 models were constructed).

In the modelling approach, the *j*th trap catch for year *k* (y_{jk}) (collected on day t_{jk}) was assumed to follow a Poisson distribution, that is,

$$y_{jk} \sim \text{Poisson}(\mu_{jk}), \tag{S.1}$$

with the expected trap catch, μ_{jk}, given by,

$$\log(\mu_{jk}) = b_0^{(k)} + \sum_{n=1}^{2} \left\{ a_n^{(k)} \sin\left(\frac{2n\pi}{365} t_{jk}\right) + b_n^{(k)} \cos\left(\frac{2n\pi}{365} t_{jk}\right) \right\} + \varepsilon_{jk}. \tag{S.2}$$

The summation in equation (S.2) describes the seasonality in activity (with year-specific parameters $b_0^{(k)}, a_n^{(k)}$ and $b_n^{(k)}$), while ε_{jk} allows for temporal autocorrelation between observations. Inter-annual variation was incorporated by assuming the parameters for each year are drawn from higher-level distributions, so that,

$$b_0^{(k)} \sim \text{N}(\mu_{b_0}, \sigma_{b_0}^2),$$
$$a_n^{(k)} \sim \text{N}(\mu_{a_n}, \sigma_{a_n}^2),$$
$$b_n^{(k)} \sim \text{N}(\mu_{b_n}, \sigma_{b_n}^2),$$

Temporal autocorrelation was described by a stationary AR(1) process (Diggle *et al.* 2002), so that,

$$\begin{aligned} \varepsilon_{jk} &= \rho \varepsilon_{j-1,k} + z_{jk}, \\ z_{jk} &\sim \text{N}(0, \sigma_\varepsilon^2). \end{aligned} \tag{S.3}$$

This approach implicitly assumes the observations are regularly spaced, though because of missing data they are not quite so.

S2.2 Parameter estimation

Parameters were estimated in a Bayesian framework, implemented using OpenBUGS (version 3.2.3; http://www.openbugs.net/). Non-informative prior distributions were used for the higher-order parameters: diffuse Normal for the μs; diffuse exponential for the σs; and uniform for ρ. Convergence of the chains was assessed visually and using the Gelman-Rubin statistic implemented in OpenBUGS.

S2.3 Model checking

Models for each species/group were assessed using posterior predictive checking (Gelman *et al*. 2004). More specifically, the posterior predictive distribution was used to generate replicated data by sampling parameter sets from the posterior distribution and using the sampled parameters to simulate data-sets using the model, (S.1)-(S.3). These were compared to the observed data using three measures: (i) χ^2 goodness-of-fit statistic (as a measure of overall fit); (ii) total catch; and (iii) maximum daily catch. If the observed data generate a more extreme value of the measures than the replicate data (as judged by the proportion of replicates which generate a value of the measure less than the observed data; this is equivalent to a classical (i.e. non-Bayesian) *P*-value), this provides an indication that the model does not adequately capture the data. In addition, the proportion of observed catches that lie outside the 95% prediction interval for the model was computed.

S2.4 Posterior inference for phenology and abundance measures

Linear regression was used to explore annual trends by fitting a straight-line relationship to each replicated measure of phenology or abundance (note: abundance measures were log transformed) and testing the significance of the slope. This generates a posterior distribution of regression coefficients and *P*-values (the latter are analogous to classical *P*-values; Meng 1994) which can be used to determine whether or not the trend in a measure is significant in a way that is robust to uncertainty in a measure.

Annual trends in the five model parameters (see equation (S.2)) were assessed by fitting a straight-line relationship to the parameter for each year and testing the significance of the slope. As before, this generates a posterior distribution of regression coefficients and *P*-values

The relationship between the five phenology and abundance measures and climate was assessed for each climate variable and the density of two livestock species shown in Table S1. Linear regression was used to fit a straight line relationship between each replicated measure

(as before, abundance measures were log transformed) and the variable of interest and testing the significance of the slope. Again, this was used to generate a posterior distribution of regression coefficients and *P*-values.

Subsequently, multiple regression was carried out using a model including all those variables for which there was a significant univariable relationship with a measure. Model selection started from a model including all variables and proceeded by stepwise deletion of non-significant (P>0.05) terms. Again, this was done using the replicated simulated measures to assess the robustness of any relationships.

S2.5 Statistical models for **Culicoides** *activity*

The models for individual species/groups are shown in figures S5-S15 for Preston and figures S16-S26 for Starcross. Posterior predictive checking indicated that all the models adequately captured the data for all three measures considered (χ^2 goodness-of-fit, total catch and maximum daily catch) and in terms of the proportion of observed catches that lie outside the 95% prediction intervals.

References for Appendices S1 & S2

Diggle, P.J., Heagarty, P., Liang, K.Y. & Zeger, S.L. (2002) *Analysis of longitudinal data, 2nd Edn.*, Oxford: Oxford University Press.

Fuller, R.M., Groom, G.B. & Jones, A.R. (1994) The land-cover map of Great Britain - an automated classification of landsat thematic mapper data. *Photogrammetric Engineering and Remote Sensing*, **60**, 553-562.

Fuller, R.M., Smith, G.M., Sanderson, J.M., Hill, R.A. & Thomson, A.G. (2002) The UK Land Cover Map 2000: Construction of a parcel-based vector map from satellite images. *Cartographic Journal* **39**, 15-25.

Gelman, A., Carlin, J.B., Stern, H.S. & Rubin, D.B. (2004) *Bayesian data analysis*. Chapman & Hall/CRC.

Meng, X.L. (1994) Posterior predictive p-values. *Annals of Statistics*, **22**, 1142-1160.

Morton, D. *et al.* (2011) CS Technical Report No 11/07 Final Report for LCM2007 - the new UK land cover map July 2011. Centre for Ecology & Hydrology (Natural Environment Research Council) Centre for Ecology & Hydrology (Natural Environment Research Council).

Sanders, C.J., Shortall, C., Gubbins, S., Burgin, L., Gloster, J., Harrington, R., Reynolds, D.R., Mellor, P.S. & Carpenter, S.T. (2011) Influence of season and meteorological parameters on flight activity of *Culicoides* biting midges. *Journal of Applied Ecology*, **48**, 1355-1364.

Table S1. Climate and livestock variables considered in the analysis of *Culicoides* abundance and phenology.

Variable	measure
temperature	annual mean†
	mean temperature in the preceding 30, 60, 90, 180 or 360 days‡
precipitation	annual total†
	total in the preceding 3, 6 or 12 months‡
	annual maximum number of consecutive dry days (≤0.2 mm)
	annual maximum number of consecutive wet days (≥ 10 mm)
soil moisture	annual mean†
	mean in the preceding 1, 2, 3, 6 or 12 months‡
North Atlantic oscillation	Hurrell station-based index
Livestock	number of cattle
	number of sheep

† for the time of first appearance this was the for the preceding year

‡ only for times of first and last appearance

Table S2. Summary of annual trends in measures of *Culicoides* phenology and abundance at Starcross, 1974-2012 (posterior median and 95% credible interval).

species	change (days per year)			% change per year	
	date of first appearance	date of last appearance	season length	maximum daily catch	mean daily catch
total *Culicoides*	-0.1 (-0.8, 0.3)	0.1 (-0.4, 0.5)	0.2 (-0.3, 0.5)	1.0 (-1.8, 4.0)	0.9 (-0.5, 2.3)
C. obsoletus group (♀)	-0.1 (-0.7, 0.5)	-0.1 (-0.7, 0.6)	0.0 (-0.8, 0.9)	-0.1 (-2.2, 2.0)	-0.1 (-0.8, 0.6)
C. obsoletus s.s. (♂)	†	†	†	0.2 (-2.4, 2.7)	0.0 (-0.2, 0.3)
C. scoticus (♂)	-0.1 (-1.5, 1.1)	0.1 (-1.1, 1.4)	0.3 (-1.7, 2.0)	1.4 (-2.6, 5.6)	0.4 (-0.5, 1.2)
C. chiopterus (♂)	†	†	†	-0.6 (-3.4, 2.2)	-0.1 (-0.6, 0.2)
C. dewulfi (♂)	†	†	†	0.4 (-3.1, 4.0)	0.1 (-0.5, 0.6)
C. pulicaris (♀)	-0.1 (-2.0, 1.7)	-0.1 (-2.2, 2.1)	0.1 (-2.5, 2.6)	0.0 (-3.0, 3.0)	0.0 (-0.4, 0.4)
C. pulicaris (♂)	-0.2 (-1.2, 0.7)	0.4 (-0.8, 1.6)	0.6 (-0.9, 2.3)	1.5 (-2.7, 5.7)	0.7 (-0.6, 2.2)
C. punctatus (♀)	-0.2 (-0.8, 0.5)	0.3 (-1.4, 2.1)	0.5 (-1.5, 2.5)	0.8 (-2.4, 3.9)	0.4 (-0.7, 1.5)
C. punctatus (♂)	-0.5 (-1.3, 0.0)	1.0 (-0.2, 2.4)	1.5 (0.2, 3.3)	6.2 (1.0, 11.7)	4.8 (1.8, 8.2)
other *Culicoides*	0.1 (-0.4, 0.5)	-0.2 (-1.1, 0.6)	-0.3 (-1.3, 0.7)	-1.4 (-4.3, 1.6)	-0.9 (-2.2, 0.3)

† insufficient individuals were caught to be able to define the dates of first and last appearance

Figure Legends

Figure S1. Geographical variation in the slope values for the 1974-2011 trend in (*a*) annual mean temperature and (*b*) annual total precipitation per pixel. All UK pixels show an increasing trend in annual mean temperature over the study period and that most pixels show an increasing trend that is as strong as that seen in Preston (b=0.03) and Starcross (b=0.02), with warming being most pronounced in the south and east of the UK. Most UK pixels show an increasing trend in annual total precipitation, but pixels with a strong increasing trend ($b \geq 4$), equivalent to that seen in Preston tend to be confined to Scotland, Cumbria and upland areas of North and South Wales.

Figure S2. Climate variables for Preston (red circles) and Starcross (blue circles). (*a*) Annual mean temperature (°C). (*b*) Annual total precipitation (mm). (*c*) Maximum number of consecutive dry days (≤0.2 mm). (*d*) Maximum number of consecutive wet days (≥ 10 mm). (*e*) Annual mean soil moisture (mm water/m soil). (*f*) North Atlantic oscillation (NAO) Hurrell Station-based index.

Figure S3. Number of livestock in a 10 km by 10 km grid square around Preston (red circles) and Starcross (blue circles) suction traps. Each figure shows the number of (*a*) cattle and (*b*) sheep from agricultural survey data, 1972-2010.

Figure S4. Percentage land cover of the nine aggregate types from each LCM map temporal snapshot for Starcross (left hand panel) and Preston (right hand panel) at the small (top row) and large (bottom row) buffer zone sizes.

Figure S5. Total number of *Culicoides* collected in the Rothamsted Insect Survey suction trap at Preston, 1974-2012. The red circles are the observed daily trap catches, while the blue line shows the median, the blue shading the posterior density and the black dotted lines the 2.5th and 97.5th percentiles for the posterior predictive distribution for the daily trap catch.

Figure S6. Number of *C. obsoletus* females collected in the Rothamsted Insect Survey suction trap at Preston, 1974-2012. The red circles are the observed daily trap catches, while the blue line shows the median, the blue shading the posterior density and the black dotted lines the 2.5th and 97.5th percentiles for the posterior predictive distribution for the daily trap catch.

Figure S7. Number of *C. obsoletus* males collected in the Rothamsted Insect Survey suction trap at Preston, 1974-2012. The red circles are the observed daily trap catches, while the blue line shows the median, the blue shading the posterior density and the black dotted lines the 2.5th and 97.5th percentiles for the posterior predictive distribution for the daily trap catch.

Figure S8. Number of *C. scoticus* males collected in the Rothamsted Insect Survey suction trap at Preston, 1974-2012. The red circles are the observed daily trap catches, while the blue line shows the median, the blue shading the posterior density and the black dotted lines the 2.5th and 97.5th percentiles for the posterior predictive distribution for the daily trap catch.

Figure S9. Number of *C. dewulfi* males collected in the Rothamsted Insect Survey suction trap at Preston, 1974-2012. The red circles are the observed daily trap catches, while the blue line shows the median, the blue shading the posterior density and the black dotted lines the 2.5th and 97.5th percentiles for the posterior predictive distribution for the daily trap catch.

Figure S10. Number of *C. chiopterus* males collected in the Rothamsted Insect Survey suction trap at Preston, 1974-2012. The red circles are the observed daily trap catches, while the blue line shows the median, the blue shading the posterior density and the black dotted lines the 2.5th and 97.5th percentiles for the posterior predictive distribution for the daily trap catch.

Figure S11. Number of *C. pulicaris* females collected in the Rothamsted Insect Survey suction trap at Preston, 1974-2012. The red circles are the observed daily trap catches, while the blue line shows the median, the blue shading the posterior density and the black dotted lines the 2.5th and 97.5th percentiles for the posterior predictive distribution for the daily trap catch.

Figure S12. Number of *C. pulicaris* males collected in the Rothamsted Insect Survey suction trap at Preston, 1974-2012. The red circles are the observed daily trap catches, while the blue line shows the median, the blue shading the posterior density and the black dotted lines the 2.5th and 97.5th percentiles for the posterior predictive distribution for the daily trap catch.

Figure S13. Number of *C. punctatus* females collected in the Rothamsted Insect Survey suction trap at Preston, 1974-2012. The red circles are the observed daily trap catches, while the blue line shows the median, the blue shading the posterior density and the black dotted lines the 2.5th and 97.5th percentiles for the posterior predictive distribution for the daily trap catch.

Figure S14. Number of *C. punctatus* males collected in the Rothamsted Insect Survey suction trap at Preston, 1974-2012. The red circles are the observed daily trap catches, while the blue line shows the median, the blue shading the posterior density and the black dotted lines the 2.5th and 97.5th percentiles for the posterior predictive distribution for the daily trap catch.

Figure S15. Number of other *Culicoides* collected in the Rothamsted Insect Survey suction trap at Preston, 1974-2012. The red circles are the observed daily trap catches, while the blue line shows the median, the blue shading the posterior density and the black dotted lines the 2.5th and 97.5th percentiles for the posterior predictive distribution for the daily trap catch.

Figure S16. Total number of *Culicoides* collected in the Rothamsted Insect Survey suction trap at Starcross, 1974-2012. The red circles are the observed daily trap catches, while the blue line shows the median, the blue shading the posterior density and the black dotted lines the 2.5th and 97.5th percentiles for the posterior predictive distribution for the daily trap catch.

Figure S17. Number of *C. obsoletus* females collected in the Rothamsted Insect Survey suction trap at Starcross, 1974-2012. The red circles are the observed daily trap catches, while the blue line shows the median, the blue shading the posterior density and the black dotted lines the 2.5th and 97.5th percentiles for the posterior predictive distribution for the daily trap catch.

Figure S18. Number of *C. obsoletus* males collected in the Rothamsted Insect Survey suction trap at Starcross, 1974-2012. The red circles are the observed daily trap catches, while the blue line shows the median, the blue shading the posterior density and the black dotted lines the 2.5th and 97.5th percentiles for the posterior predictive distribution for the daily trap catch.

Figure S19. Number of *C. scoticus* males collected in the Rothamsted Insect Survey suction trap at Starcross, 1974-2012. The red circles are the observed daily trap catches, while the blue line shows the median, the blue shading the posterior density and the black dotted lines the 2.5th and 97.5th percentiles for the posterior predictive distribution for the daily trap catch.

Figure S20. Number of *C. dewulfi* males collected in the Rothamsted Insect Survey suction trap at Starcross, 1974-2012. The red circles are the observed daily trap catches, while the blue

line shows the median, the blue shading the posterior density and the black dotted lines the 2.5th and 97.5th percentiles for the posterior predictive distribution for the daily trap catch.

Figure S21. Number of *C. chiopterus* males collected in the Rothamsted Insect Survey suction trap at Starcross, 1974-2012. The red circles are the observed daily trap catches, while the blue line shows the median, the blue shading the posterior density and the black dotted lines the 2.5th and 97.5th percentiles for the posterior predictive distribution for the daily trap catch.

Figure S22. Number of *C. pulicaris* females collected in the Rothamsted Insect Survey suction trap at Starcross, 1974-2012. The red circles are the observed daily trap catches, while the blue line shows the median, the blue shading the posterior density and the black dotted lines the 2.5th and 97.5th percentiles for the posterior predictive distribution for the daily trap catch.

Figure S23. Number of *C. pulicaris* males collected in the Rothamsted Insect Survey suction trap at Starcross, 1974-2012. The red circles are the observed daily trap catches, while the blue line shows the median, the blue shading the posterior density and the black dotted lines the 2.5th and 97.5th percentiles for the posterior predictive distribution for the daily trap catch.

Figure S24. Number of *C. punctatus* females collected in the Rothamsted Insect Survey suction trap at Starcross, 1974-2012. The red circles are the observed daily trap catches, while the blue line shows the median, the blue shading the posterior density and the black dotted lines the 2.5th and 97.5th percentiles for the posterior predictive distribution for the daily trap catch.

Figure S25. Number of *C. punctatus* males collected in the Rothamsted Insect Survey suction trap at Starcross, 1974-2012. The red circles are the observed daily trap catches, while the blue line shows the median, the blue shading the posterior density and the black dotted lines the 2.5th and 97.5th percentiles for the posterior predictive distribution for the daily trap catch.

Figure S26. Number of other *Culicoides* collected in the Rothamsted Insect Survey suction trap at Starcross, 1974-2012. The red circles are the observed daily trap catches, while the blue line shows the median, the blue shading the posterior density and the black dotted lines the 2.5th and 97.5th percentiles for the posterior predictive distribution for the daily trap catch.

Figure S27. Annual trends in *Culicoides* phenology and abundance at Starcross, 1974-2012. Results are presented for five measures: day (from 1 January) of first appearance (d. first app.); day (from 1 January) of last appearance (d. last app.); season length (seas. length; in days); $\log_{10}$ maximum daily catch ($\log_{10}$ max. catch); and $\log_{10}$ mean daily catch ($\log_{10}$ mean catch). In each figure the observed measures calculated from the trap catches are shown as red circles. The simulated measures generated from the generalised linear models fitted to the RST data are shown as box and whisker plots (median: horizontal black line; interquartile range: grey box; and 2.5th and 97.5th percentiles: whiskers).

Figure S28. Annual trends in parameters in the generalised linear models for *Culicoides* trap catches at (*a*) Preston and (*b*) Starcross, 1974-2012 (see Supporting information for details). Violin plots show the posterior density (shape), median (circle) and interquartile range (line) for the regression coefficients for the trend line for each parameter. Plots are coloured red where evidence for the trend is robust (median posterior *P*-value<0.05) and blue where it is not (median posterior *P*-value>0.05).

Figure S29. Relationship between (*a*) annual mean temperature, (*b*) annual total precipitation and (*c*) annual mean soil moisture and measures of *Culicoides* phenology and abundance at Starcross, 1974-2012. Results are presented for five measures: day (from 1 January) of first appearance (d. first app.); day (from 1 January) of last appearance (d. last app.); season length (seas. length); $\log_{10}$ maximum daily catch ($\log_{10}$ max. catch); and $\log_{10}$ mean daily catch ($\log_{10}$ mean catch). Each figure shows the posterior predictive distribution for the regression coefficient in a straight-line relationship between the climate variable and the measure. Violin plots show the posterior density (shape), median (circle) and interquartile range (line) for the coefficient. Plots are coloured red where evidence for the trend is robust (median posterior *P*-value<0.05) and blue where it is not (median posterior *P*-value>0.05).

Figure S30. Summary of multiple climate trends in measures of Culicoides phenology and abundance at Preston, 1974-2012. Results are presented for five measures: day (from 1 January) of first appearance (d. first app.); day (from 1 January) of last appearance (d. last app.); season length (seas. length); $\log_{10}$ maximum daily catch ($\log_{10}$ max. catch); and $\log_{10}$ mean daily catch ($\log_{10}$ mean catch). Each figure shows the proportion of simulated replicated measures for which the final multiple regression model for the measure incorporated the specified combination of climate variables. The letters indicate the climate variables included

in the final model: annual mean temperature (T); annual total precipitation (P); and annual mean soil moisture (S).

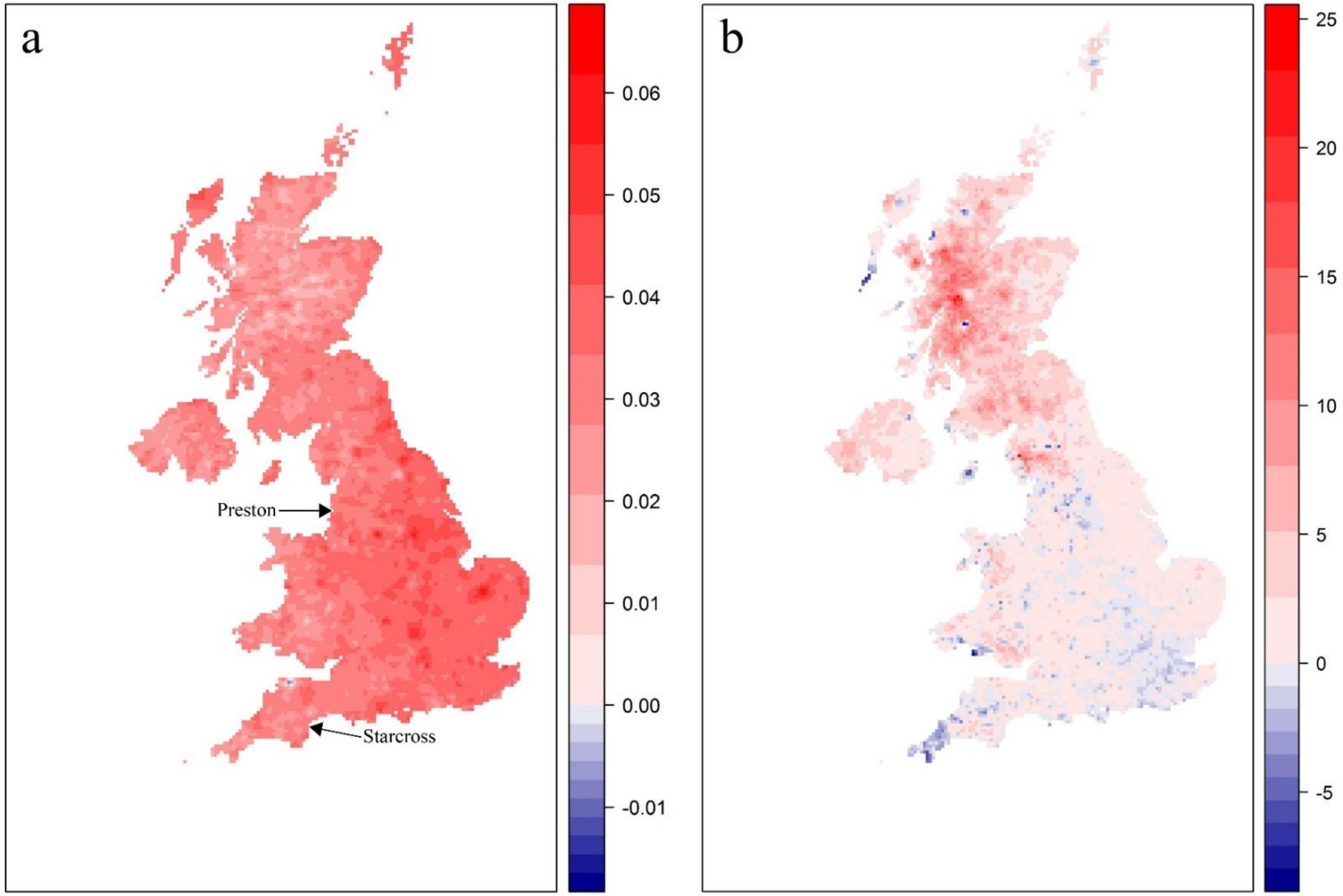
a
Preston
Starcross
0.06
0.05
0.04
0.03
0.02
0.01
0.00
-0.01
b
25
20
15
10
5
0
-5

Figure S1

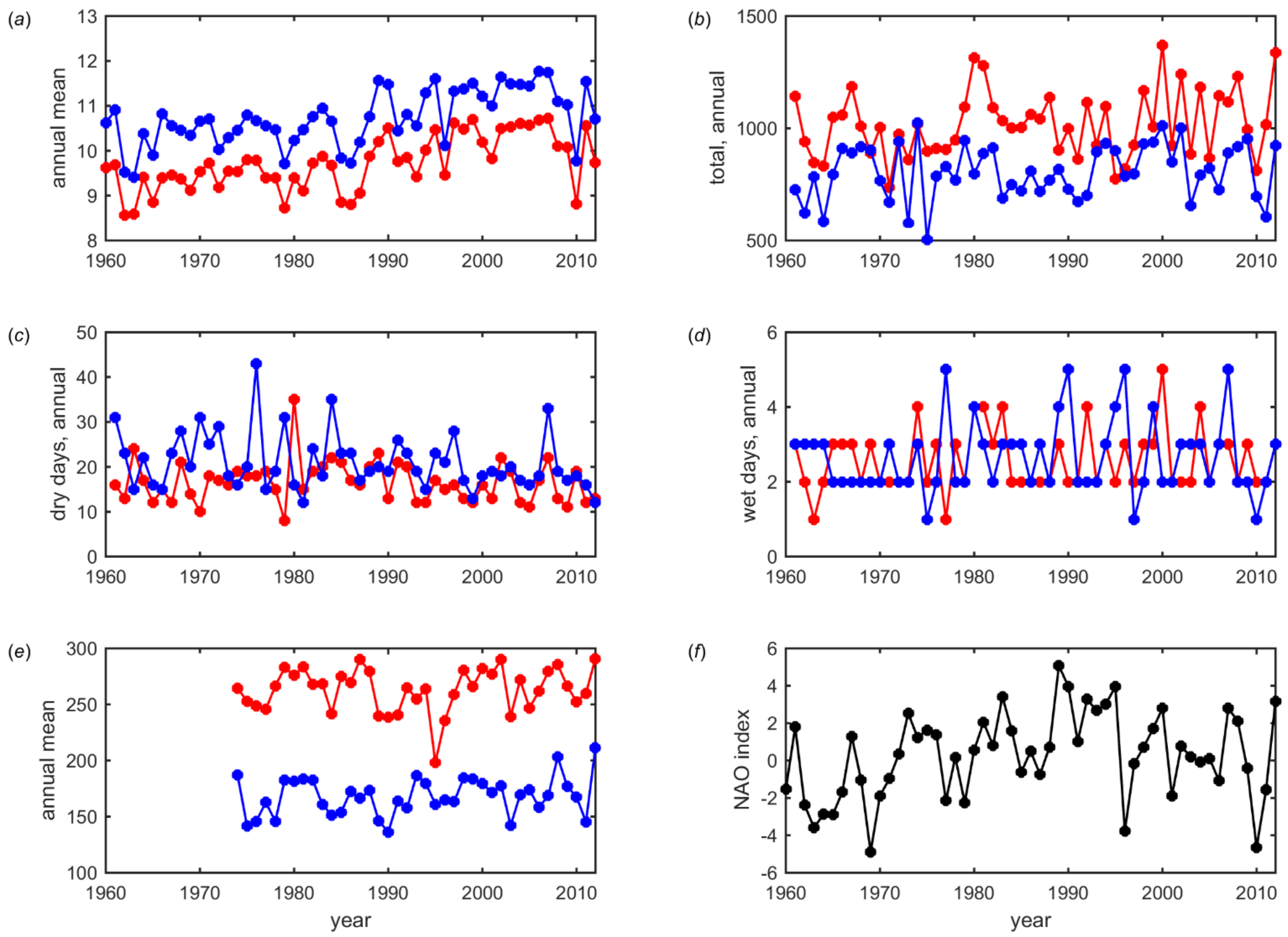
(a)
annual mean
13
12
11
10
9
8
1960
1970
1980
1990
2000
2010
(b)
total, annual
1500
1000
500
(c)
dry days, annual
50
40
30
20
10
0
(d)
wet days, annual
6
4
2
0
(e)
annual mean
300
250
200
150
100
year
(f)
NAO index
6
4
2
0
-2
-4
-6
year

Figure S2

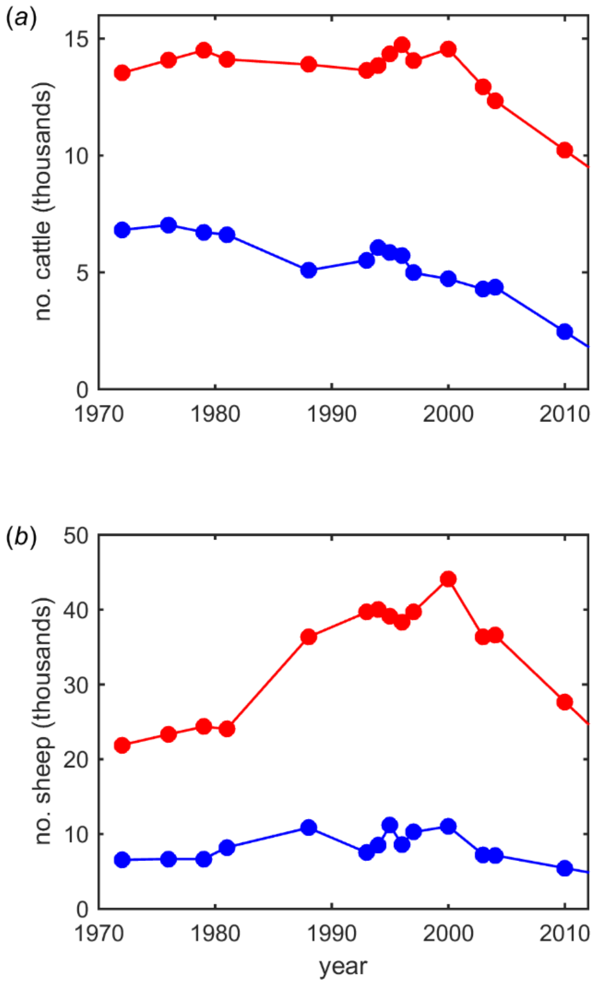
(a)
15
10
5
0
no. cattle (thousands)
1970
1980
1990
2000
2010
(b)
50
40
30
20
10
0
no. sheep (thousands)
1970
1980
1990
2000
2010
year

Figure S3

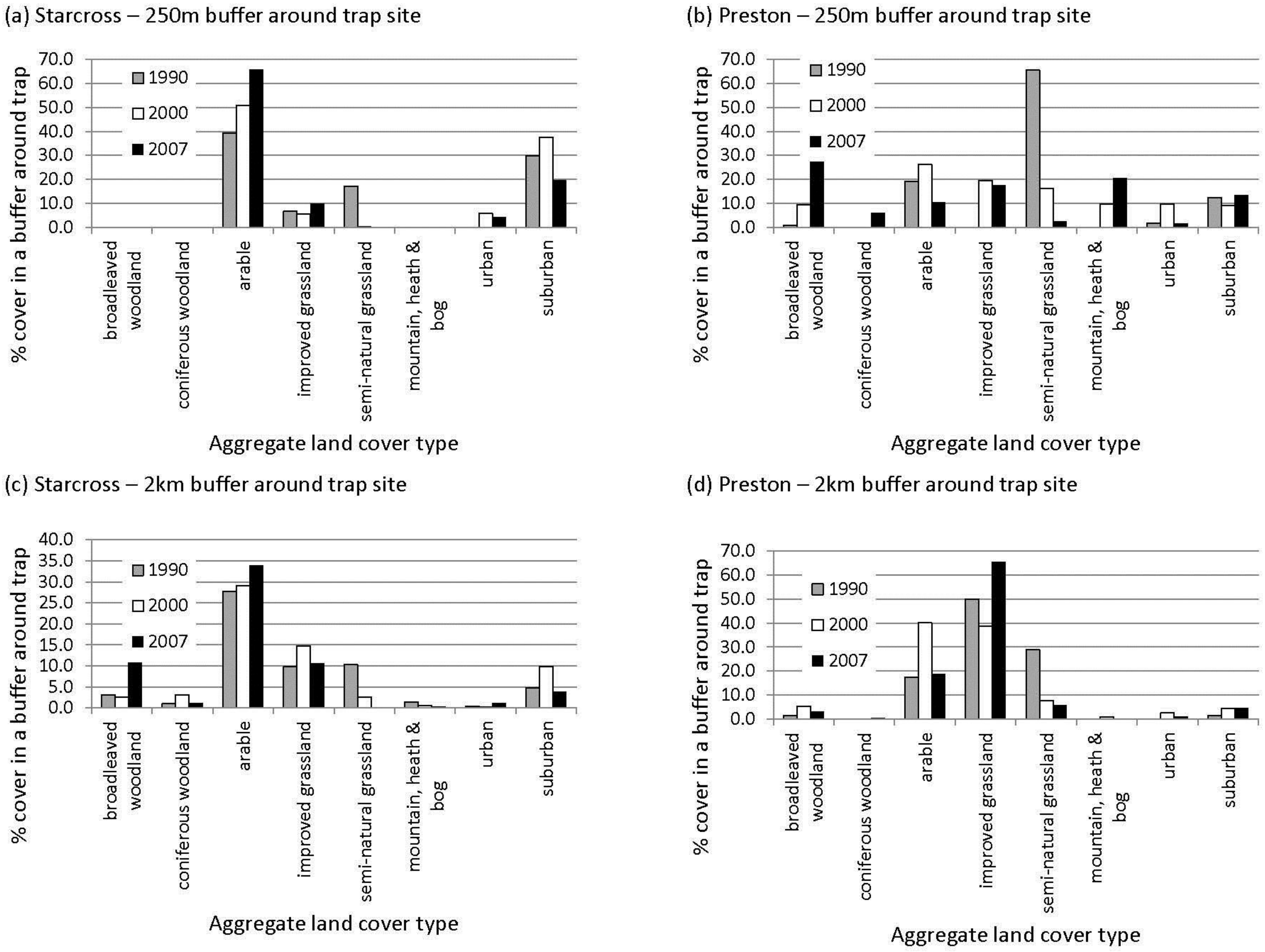
(a) Starcross – 250m buffer around trap site
(b) Preston – 250m buffer around trap site
(c) Starcross – 2km buffer around trap site
(d) Preston – 2km buffer around trap site
% cover in a buffer around trap
1990
2000
2007
broadleaved woodland
coniferous woodland
arable
improved grassland
semi-natural grassland
mountain, heath & bog
urban
suburban
Aggregate land cover type

Figure S4

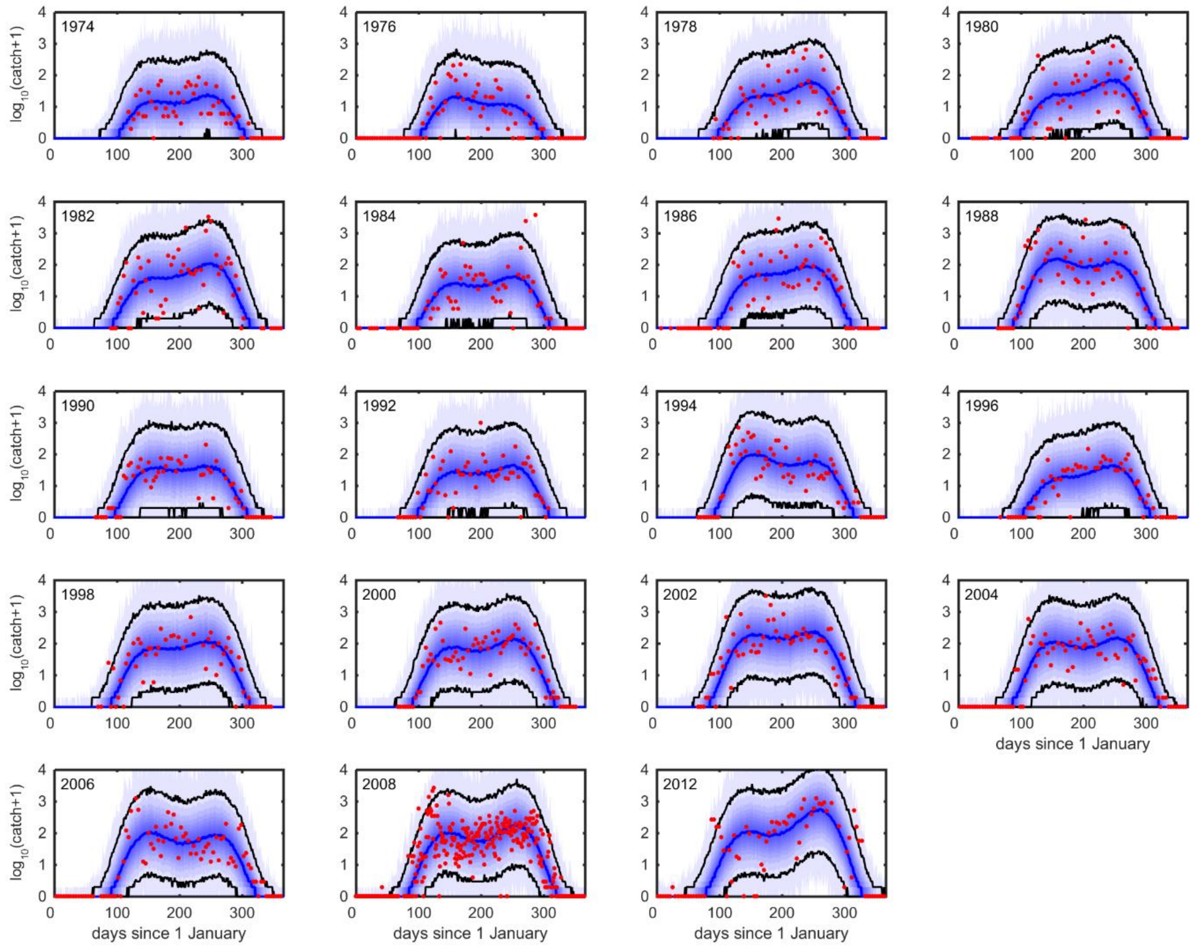
1974
1976
1978
1980
1982
1984
1986
1988
1990
1992
1994
1996
1998
2000
2002
2004
2006
2008
2012
log10(catch+1)
days since 1 January

Figure S5

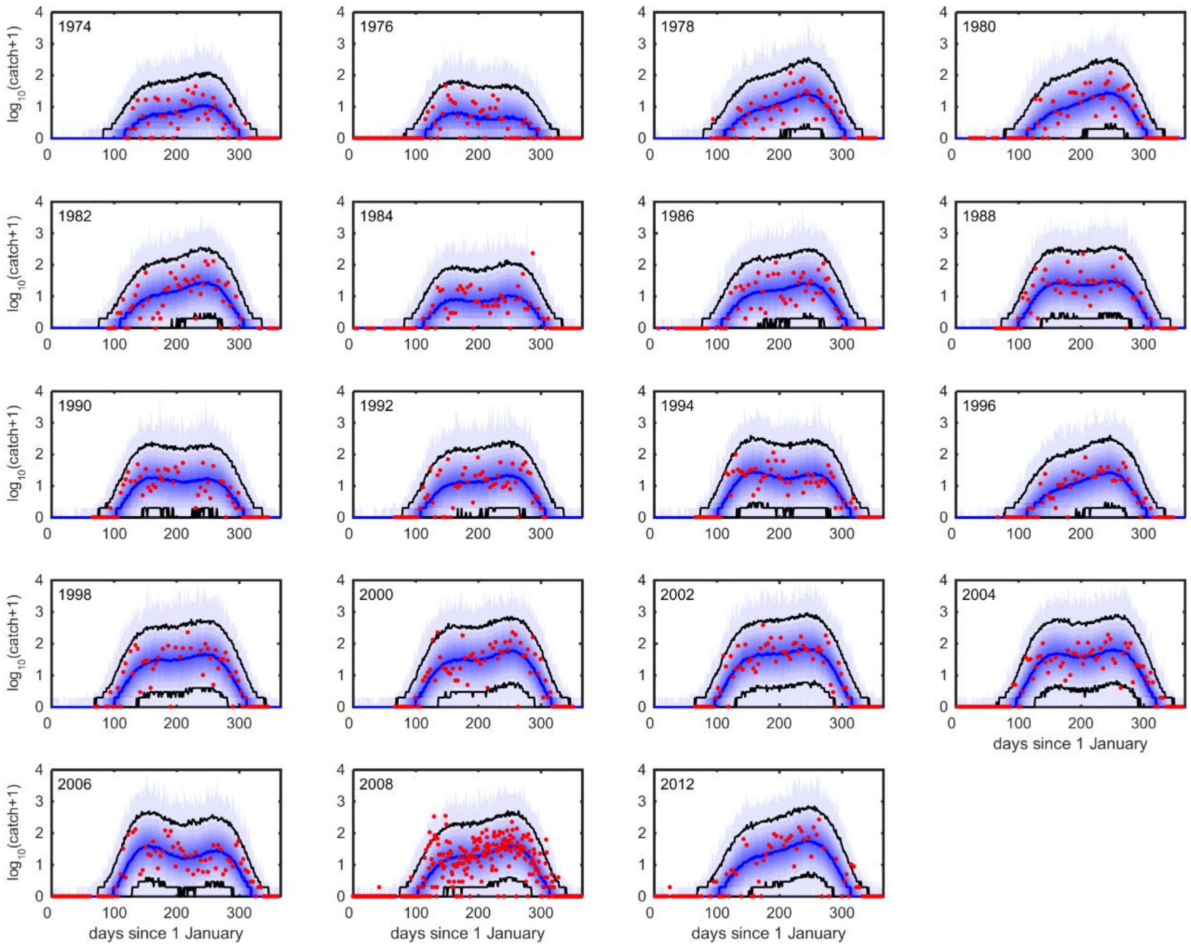
1974
1976
1978
1980
1982
1984
1986
1988
1990
1992
1994
1996
1998
2000
2002
2004
2006
2008
2012
log10(catch+1)
days since 1 January

Figure S6

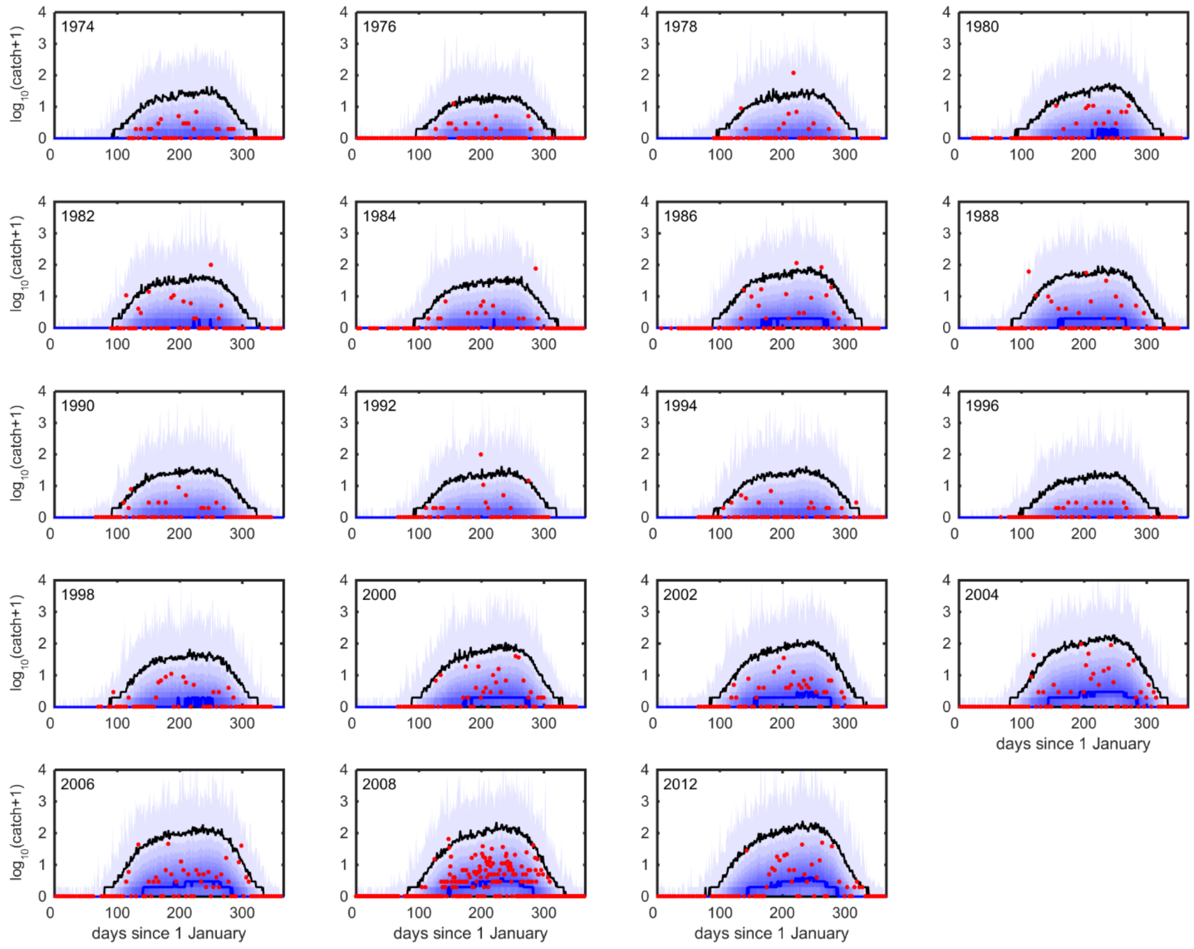

1974
1976
1978
1980
1982
1984
1986
1988
1990
1992
1994
1996
1998
2000
2002
2004
2006
2008
2012
log10(catch+1)
days since 1 January

Figure S7

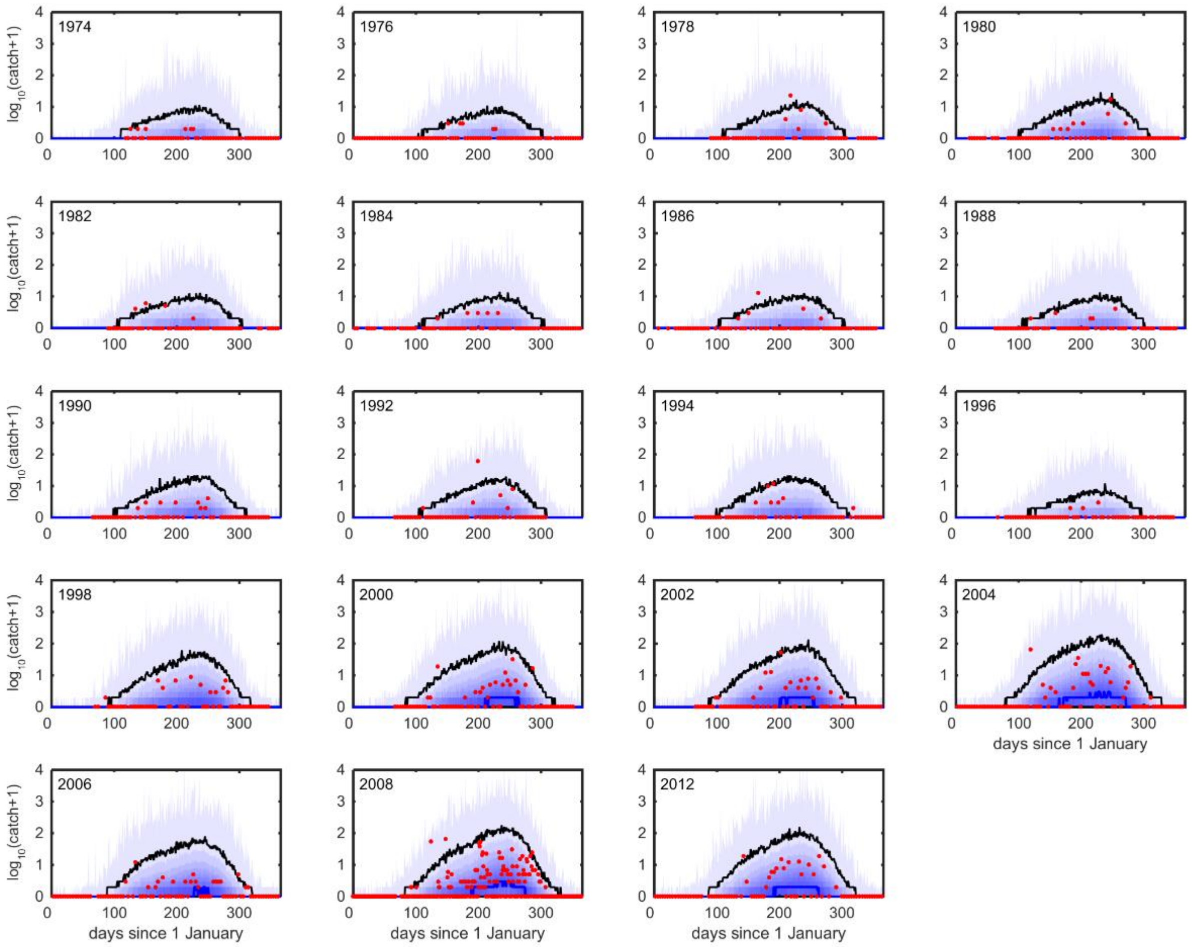
1974
1976
1978
1980
1982
1984
1986
1988
1990
1992
1994
1996
1998
2000
2002
2004
2006
2008
2012
log10(catch+1)
days since 1 January

Figure S8

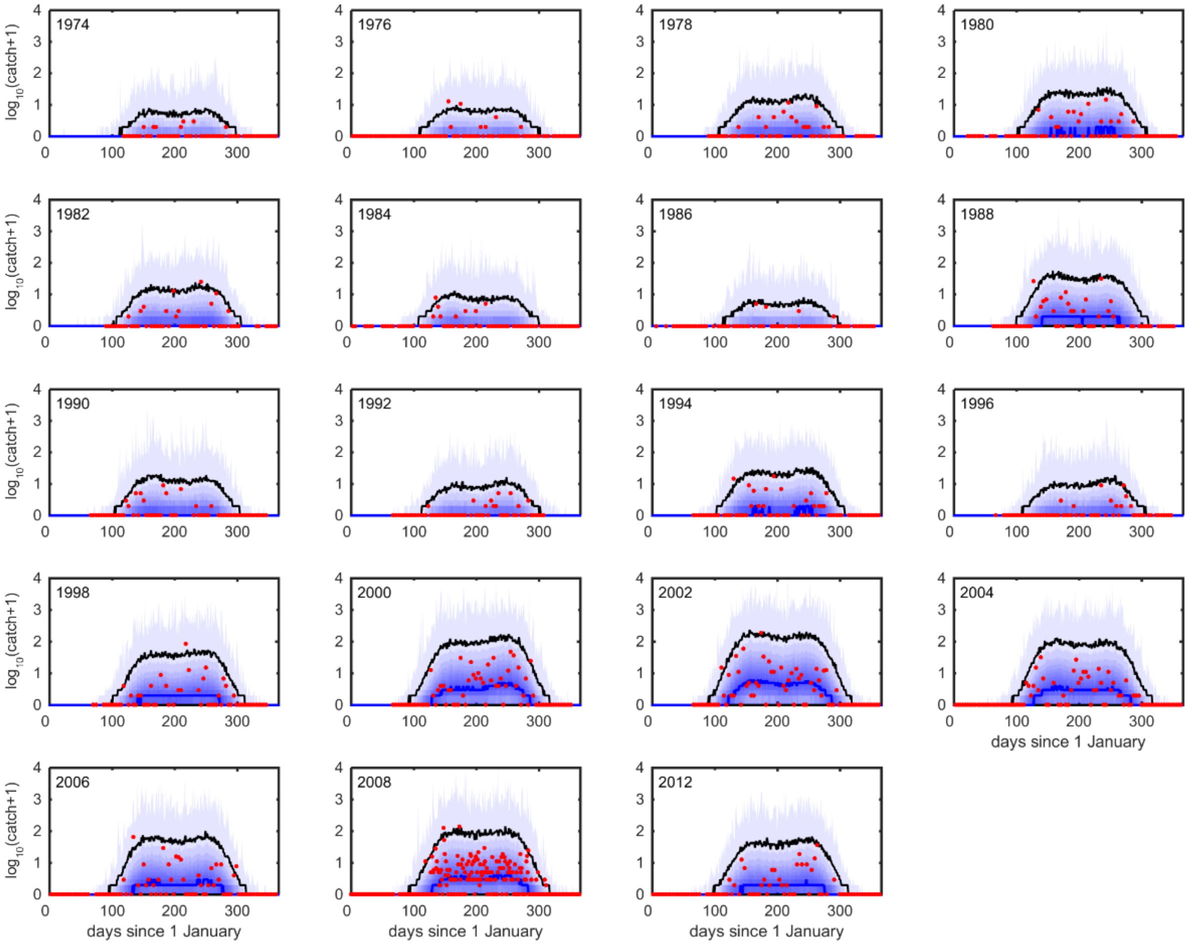
1974
1976
1978
1980
1982
1984
1986
1988
1990
1992
1994
1996
1998
2000
2002
2004
2006
2008
2012
log10(catch+1)
days since 1 January

Figure S9

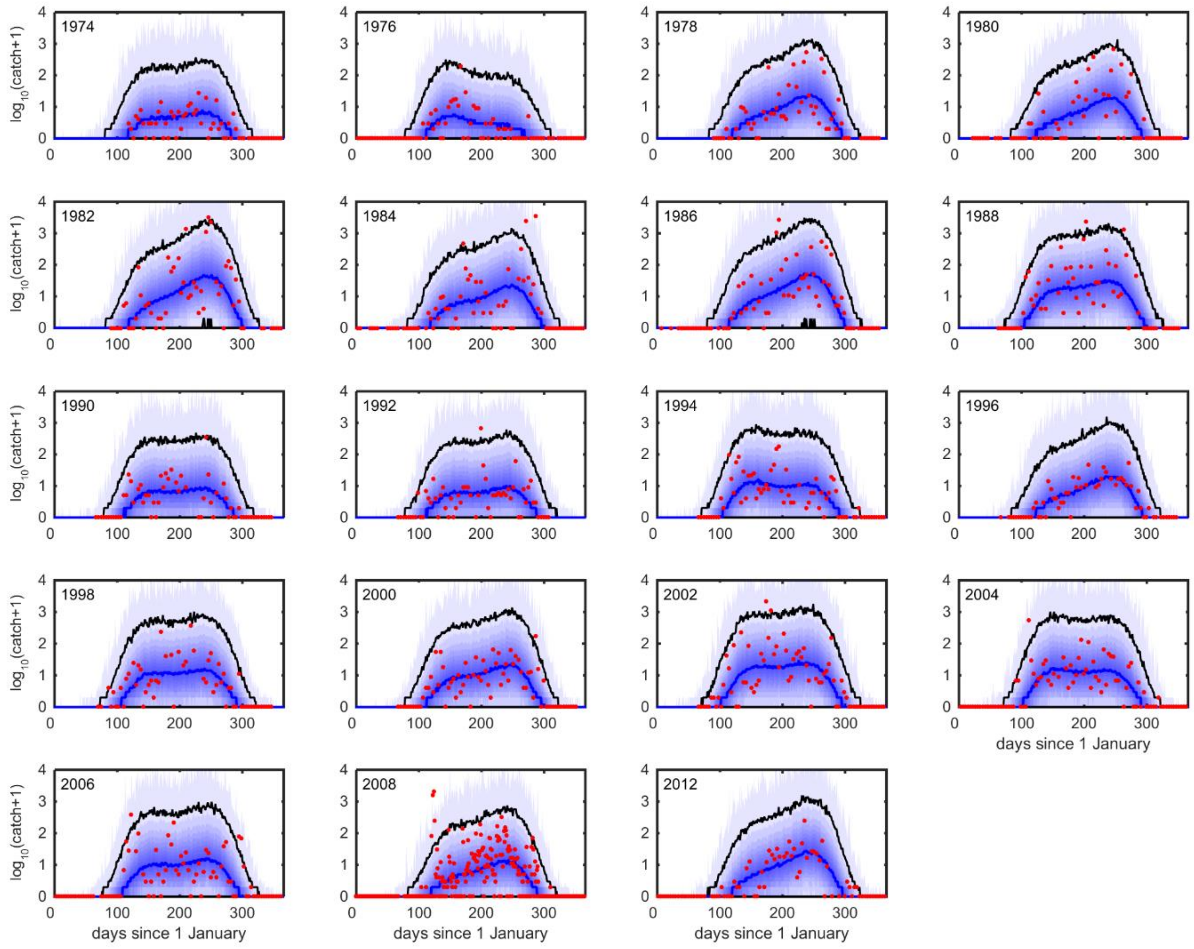
1974
1976
1978
1980
1982
1984
1986
1988
1990
1992
1994
1996
1998
2000
2002
2004
2006
2008
2012
log10(catch+1)
days since 1 January

Figure S10

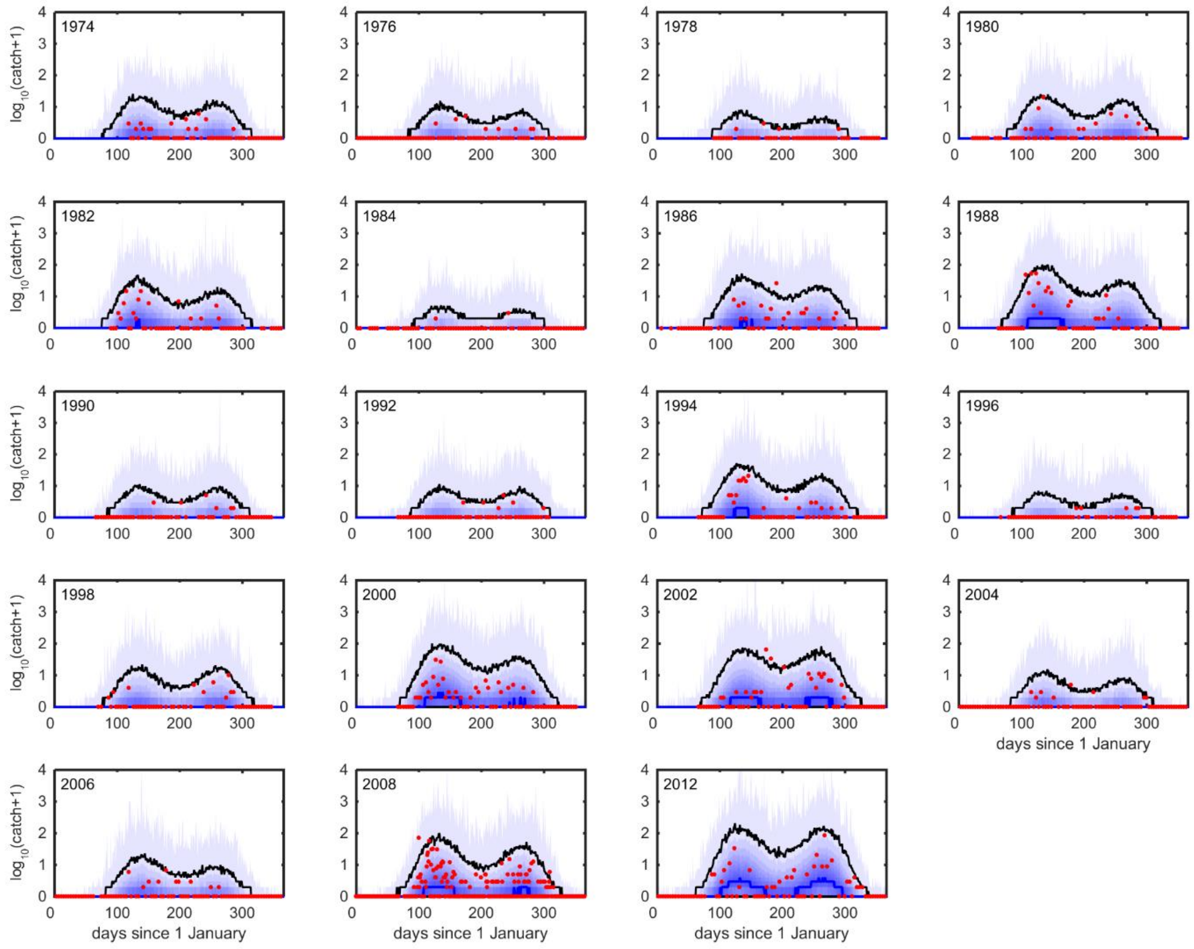
1974
1976
1978
1980
1982
1984
1986
1988
1990
1992
1994
1996
1998
2000
2002
2004
2006
2008
2012
log10(catch+1)
days since 1 January
0
100
200
300
1
2
3
4

Figure S11

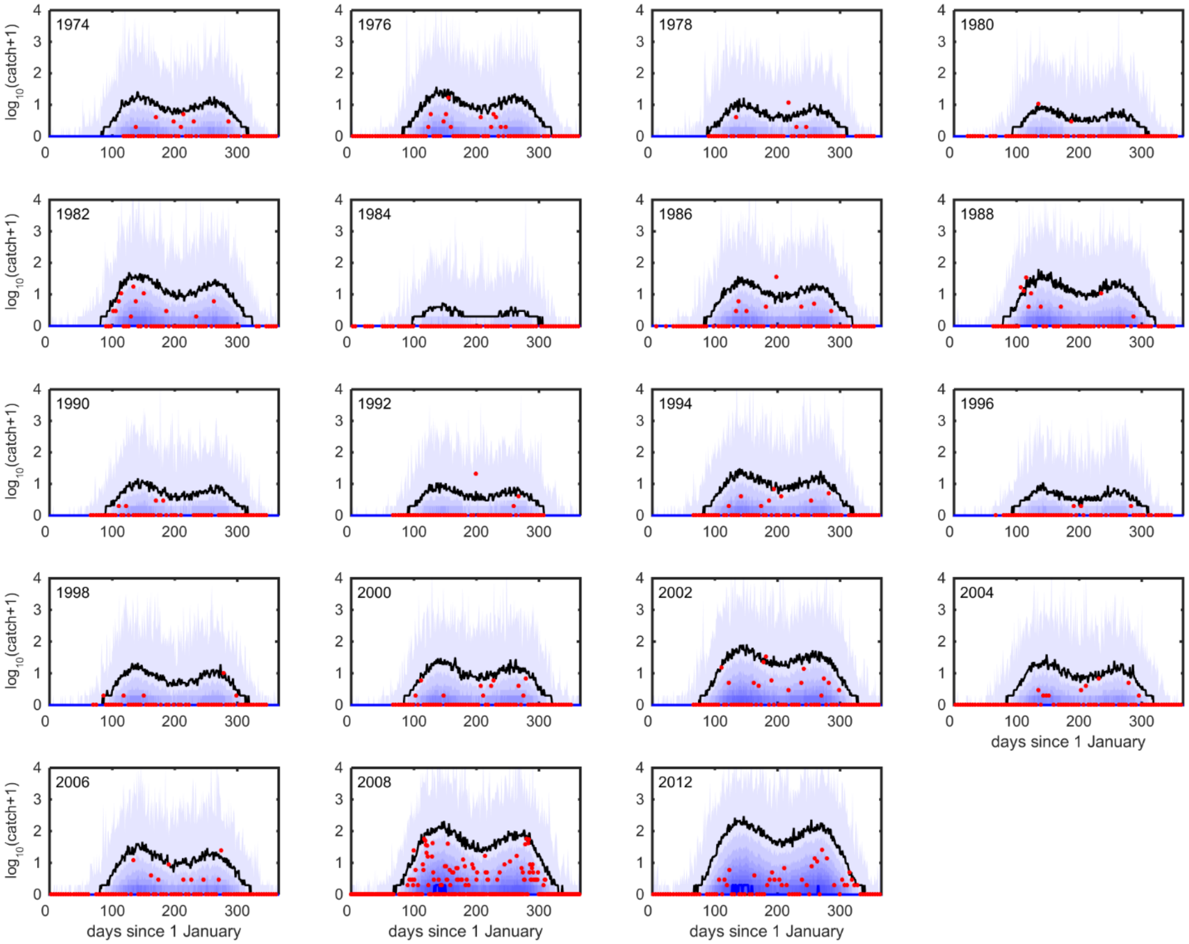

1974
1976
1978
1980
1982
1984
1986
1988
1990
1992
1994
1996
1998
2000
2002
2004
2006
2008
2012
log10(catch+1)
days since 1 January

Figure S12

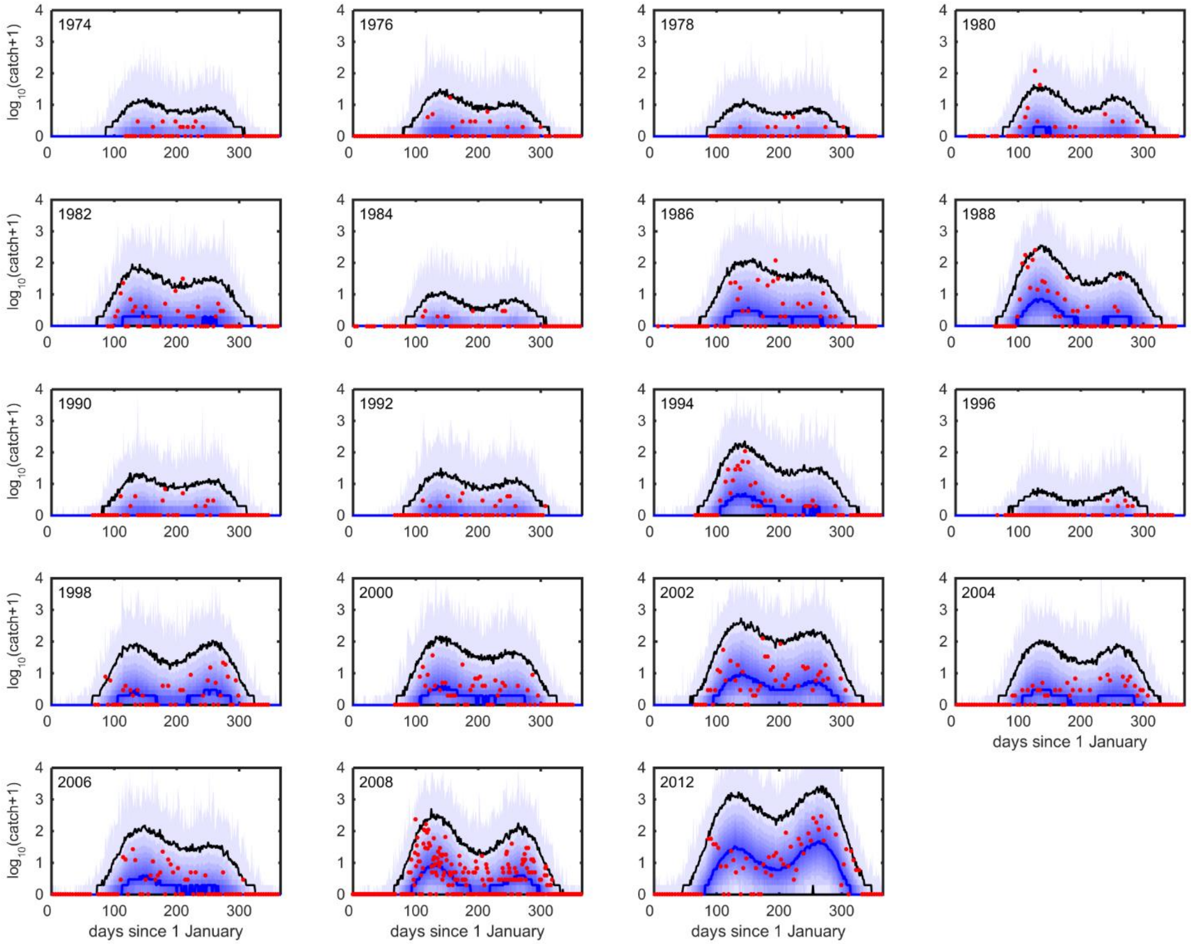
1974
1976
1978
1980
1982
1984
1986
1988
1990
1992
1994
1996
1998
2000
2002
2004
2006
2008
2012
log10(catch+1)
days since 1 January

Figure S13

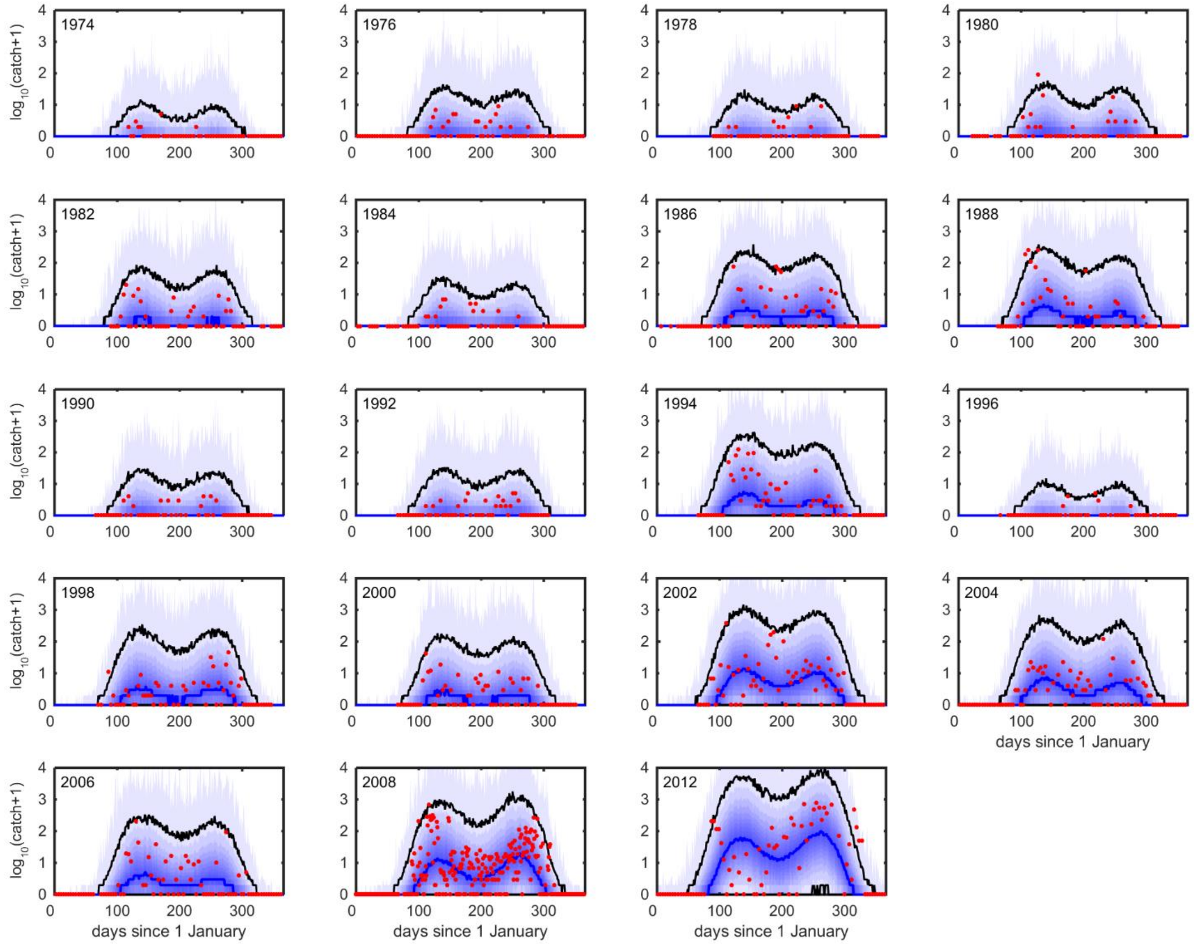
log10(catch+1)
days since 1 January
1974
1976
1978
1980
1982
1984
1986
1988
1990
1992
1994
1996
1998
2000
2002
2004
2006
2008
2012

Figure S14

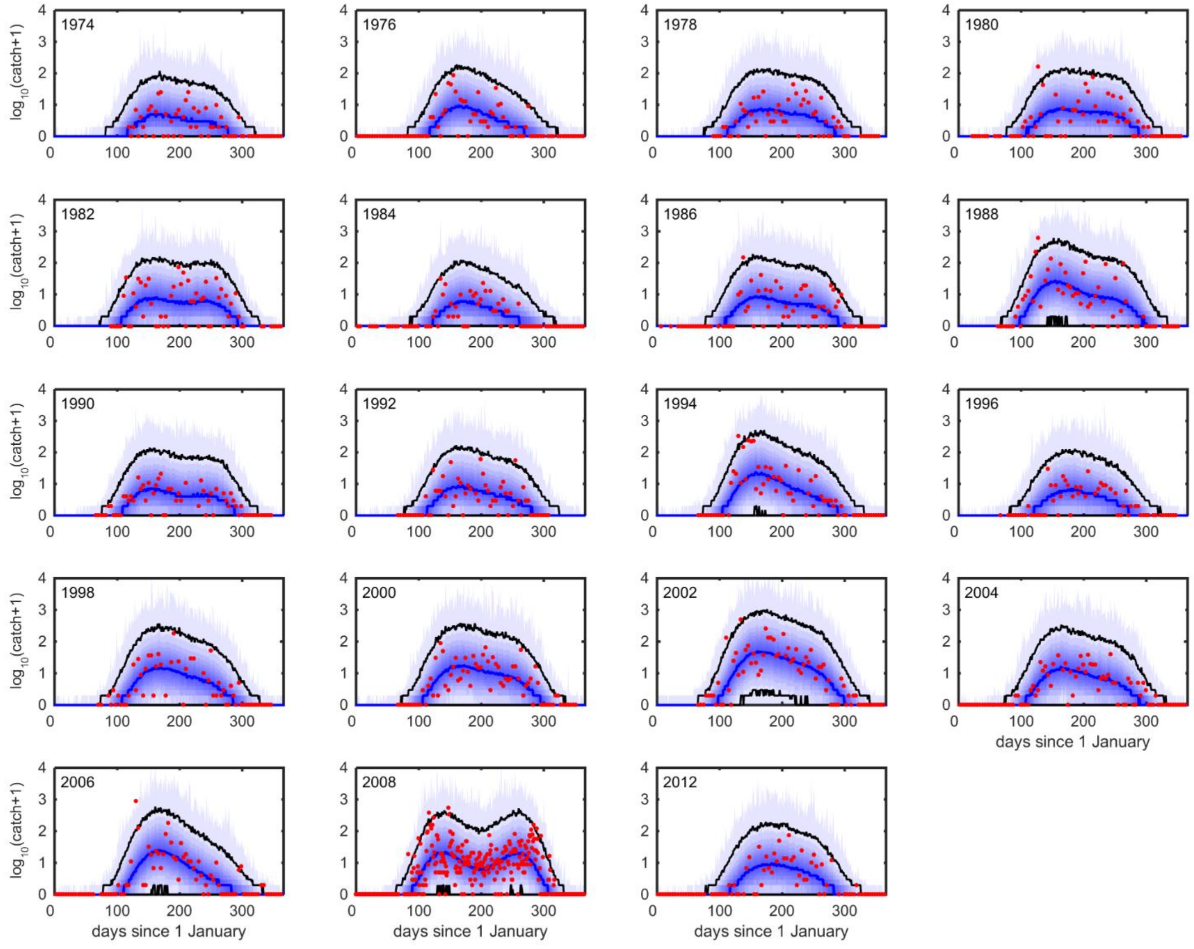

1974
1976
1978
1980
1982
1984
1986
1988
1990
1992
1994
1996
1998
2000
2002
2004
2006
2008
2012
log10(catch+1)
days since 1 January

Figure S15

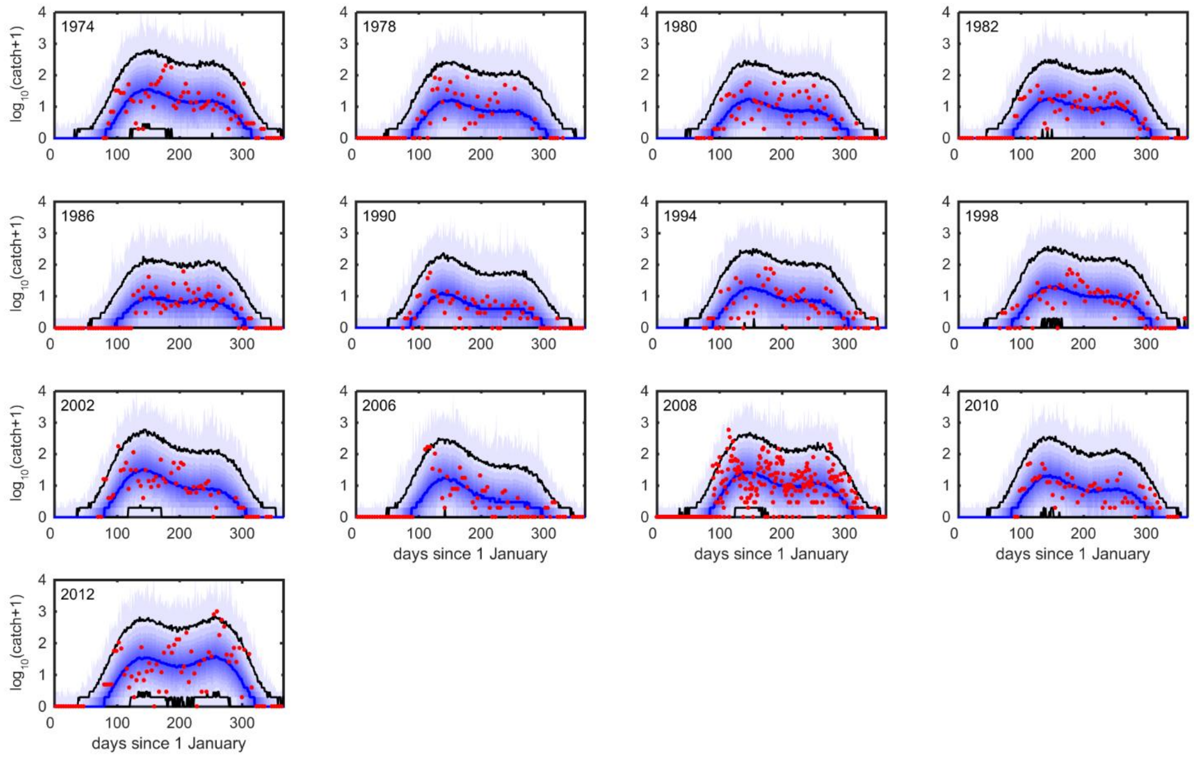
1974
1978
1980
1982
1986
1990
1994
1998
2002
2006
2008
2010
2012
log10(catch+1)
days since 1 January

Figure S16

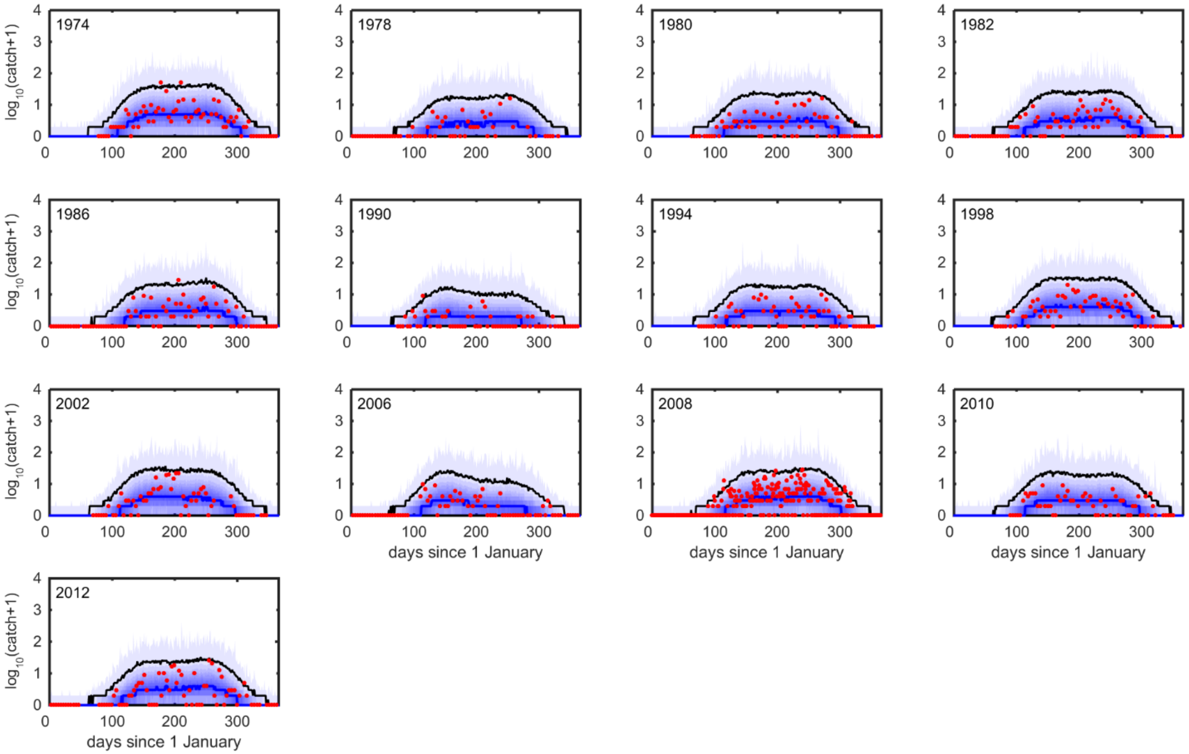
1974
1978
1980
1982
1986
1990
1994
1998
2002
2006
2008
2010
2012
log10(catch+1)
days since 1 January

Figure S17

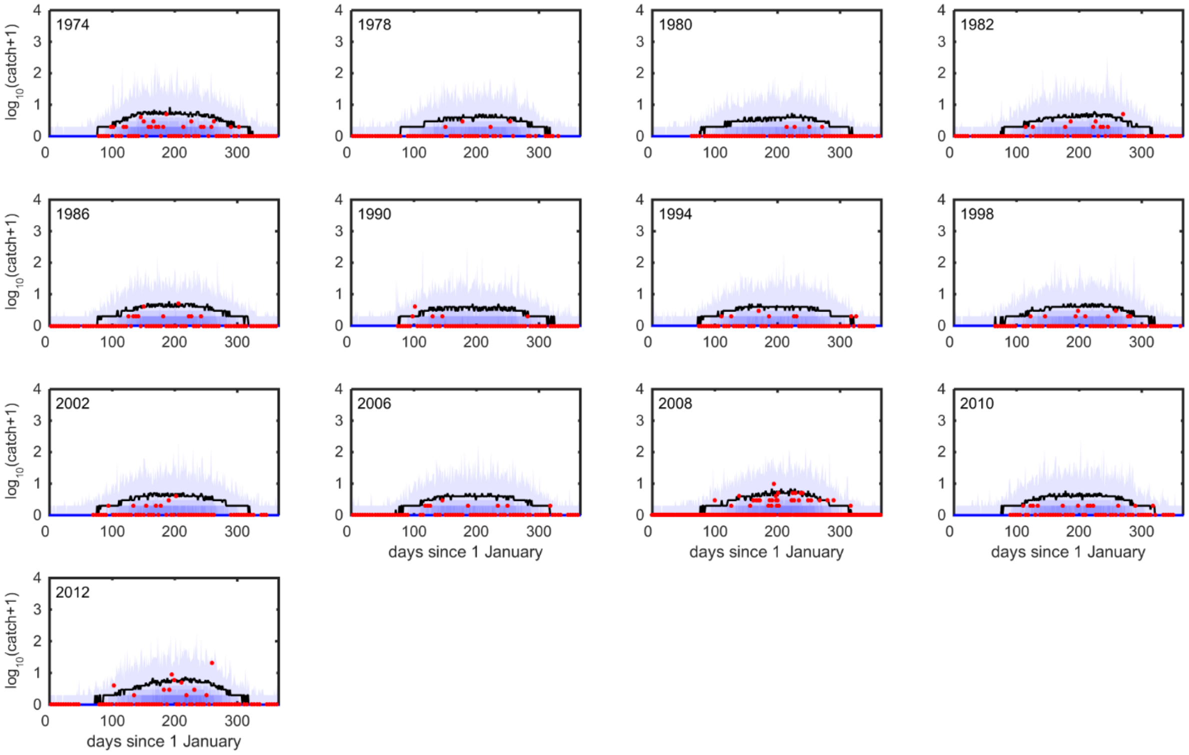
1974
1978
1980
1982
1986
1990
1994
1998
2002
2006
2008
2010
2012
log10(catch+1)
days since 1 January

Figure S18

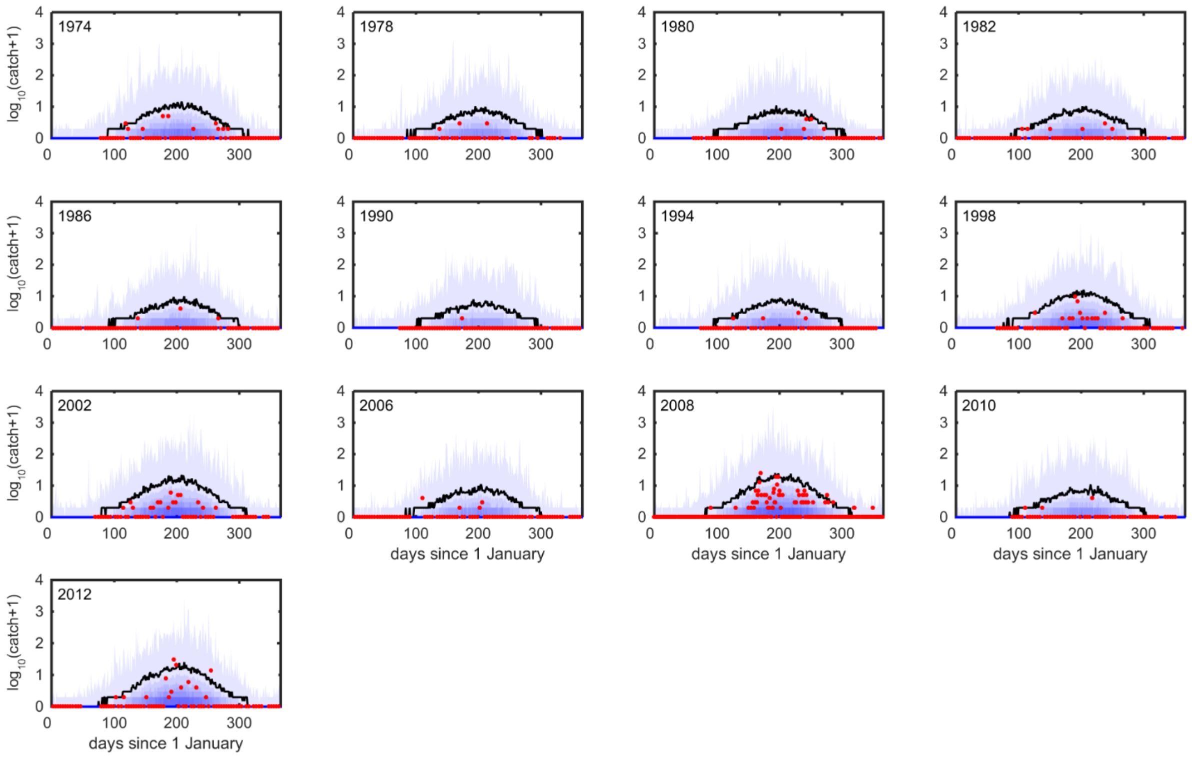

1974
1978
1980
1982
1986
1990
1994
1998
2002
2006
2008
2010
2012
log10(catch+1)
days since 1 January

Figure S19

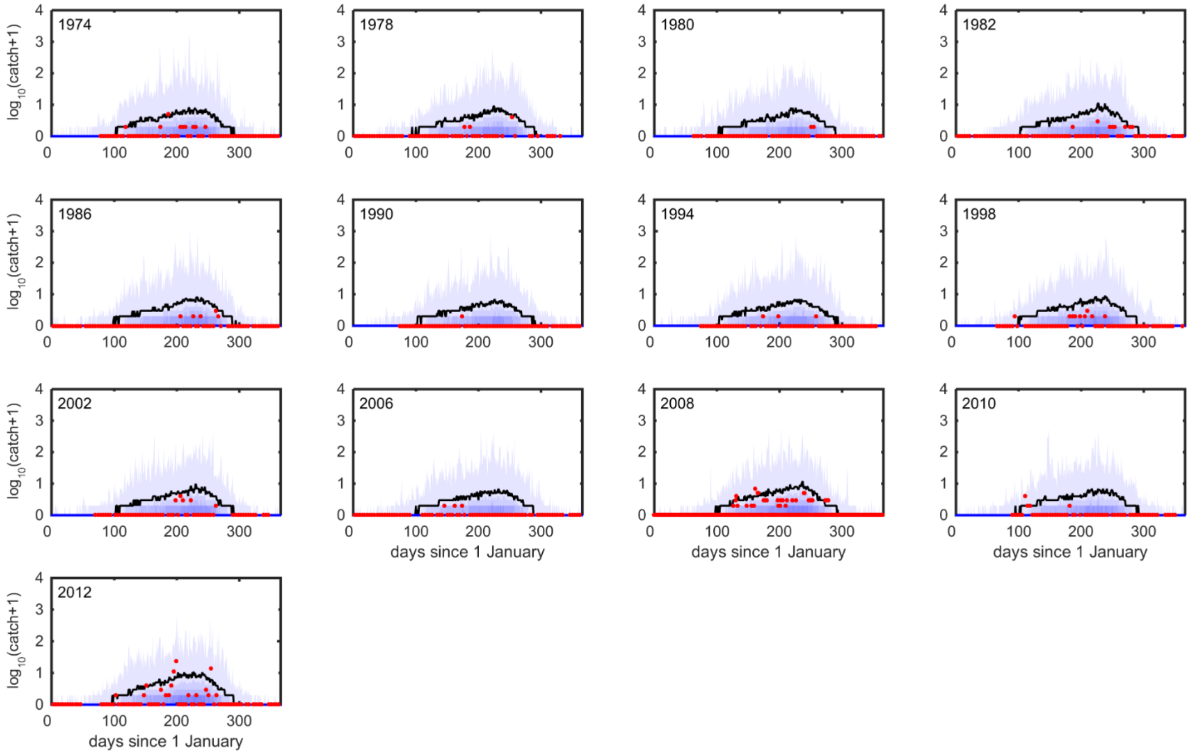

1974
1978
1980
1982
1986
1990
1994
1998
2002
2006
2008
2010
2012
log10(catch+1)
days since 1 January

Figure S20

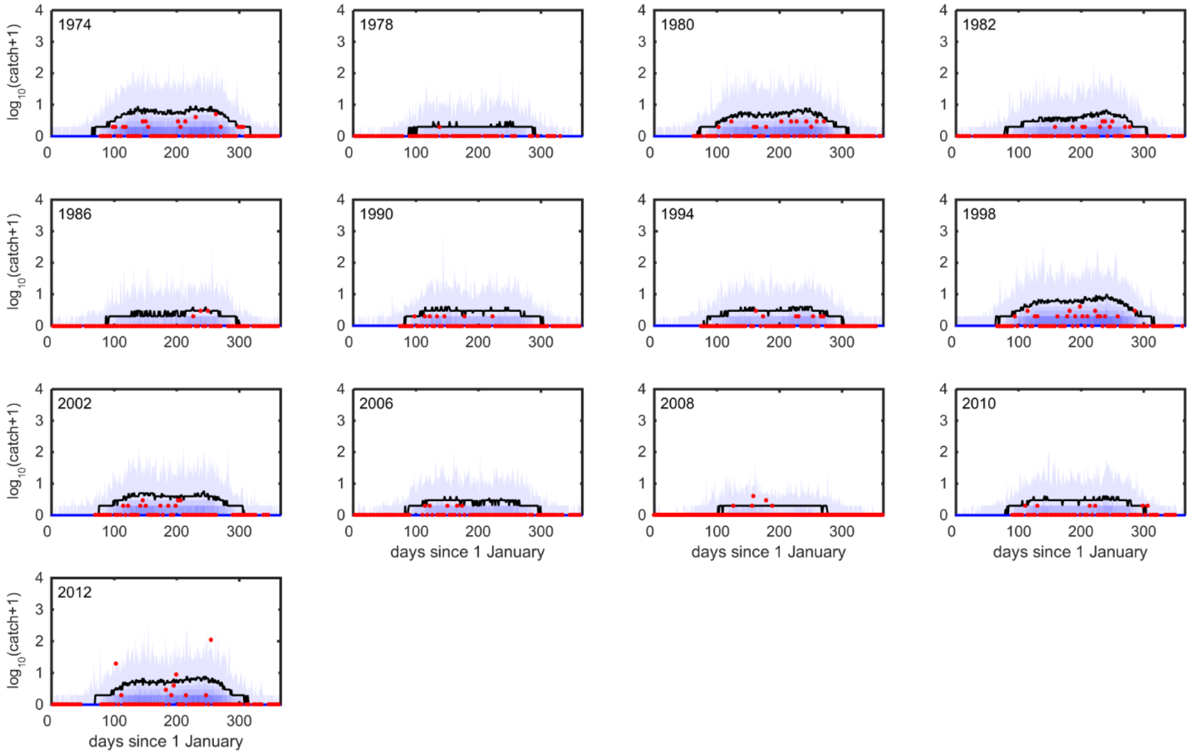
1974
1978
1980
1982
1986
1990
1994
1998
2002
2006
2008
2010
2012
log10(catch+1)
days since 1 January
0
1
2
3
4
100
200
300

Figure S21

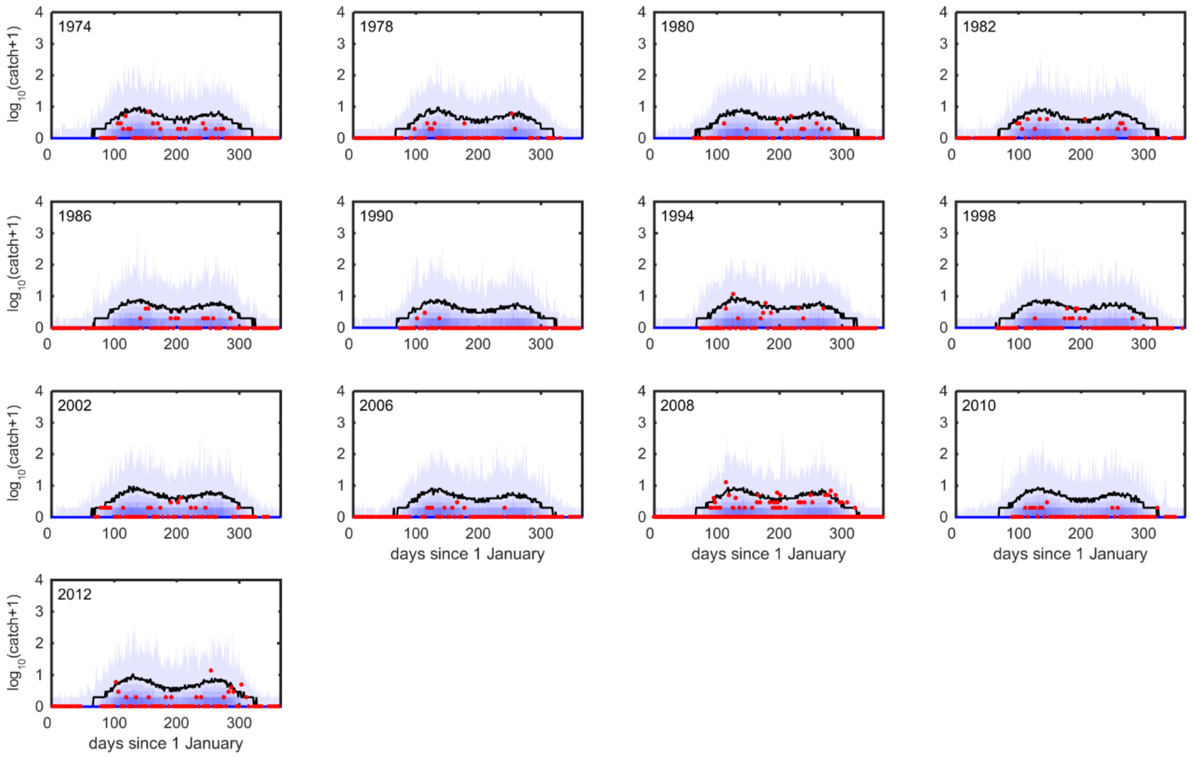
1974
1978
1980
1982
1986
1990
1994
1998
2002
2006
2008
2010
2012
log10(catch+1)
days since 1 January

Figure S22

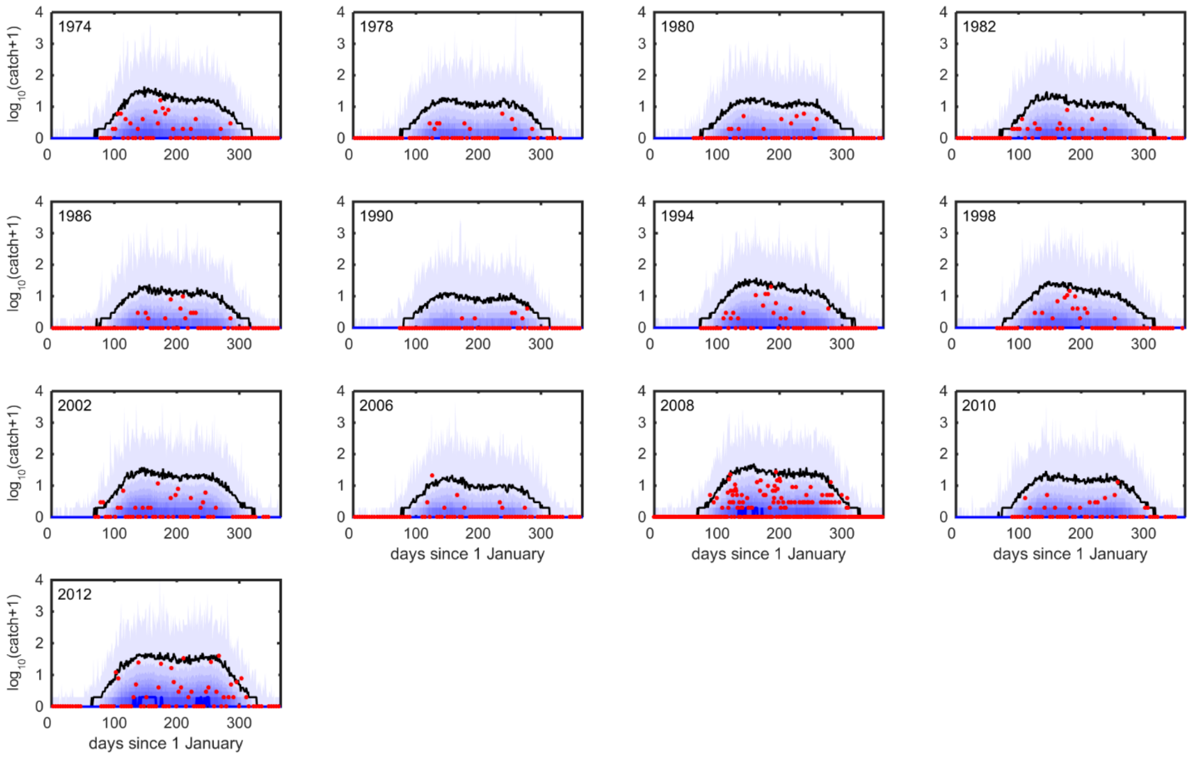
1974
1978
1980
1982
1986
1990
1994
1998
2002
2006
2008
2010
2012
log10(catch+1)
days since 1 January
0
1
2
3
4
100
200
300

Figure S23

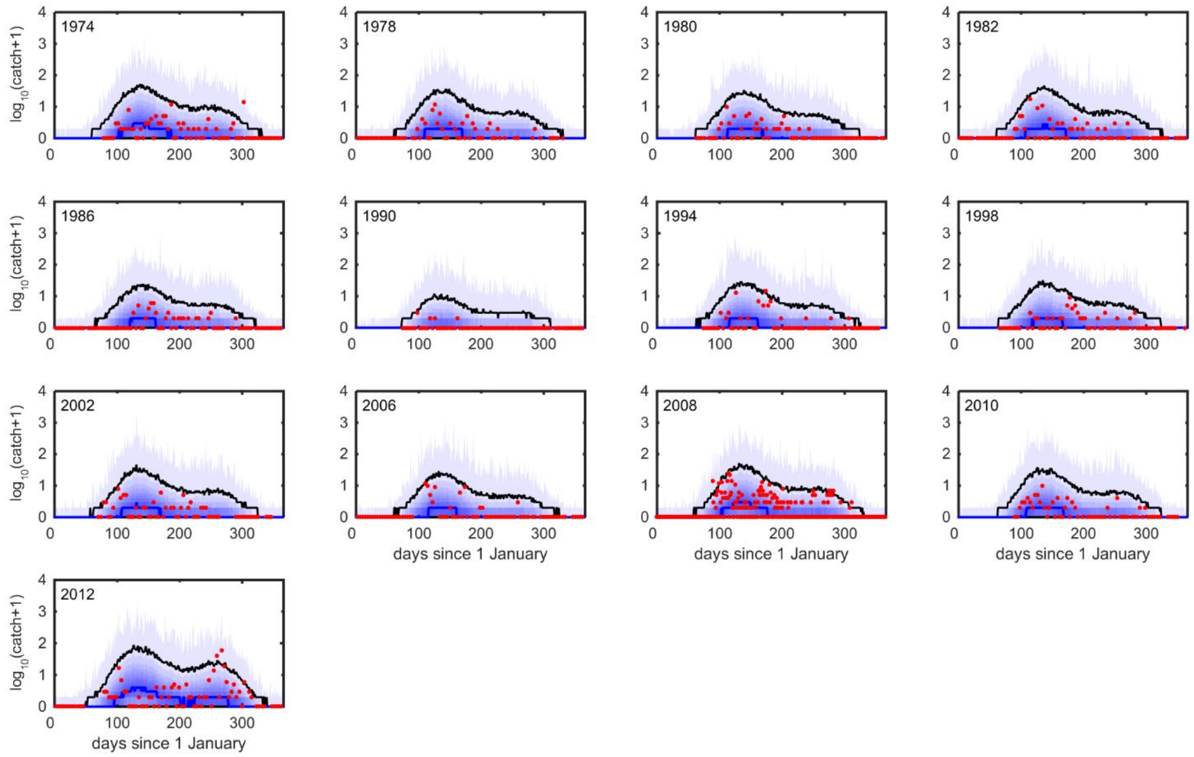
1974
1978
1980
1982
1986
1990
1994
1998
2002
2006
2008
2010
2012
log10(catch+1)
days since 1 January

Figure S24

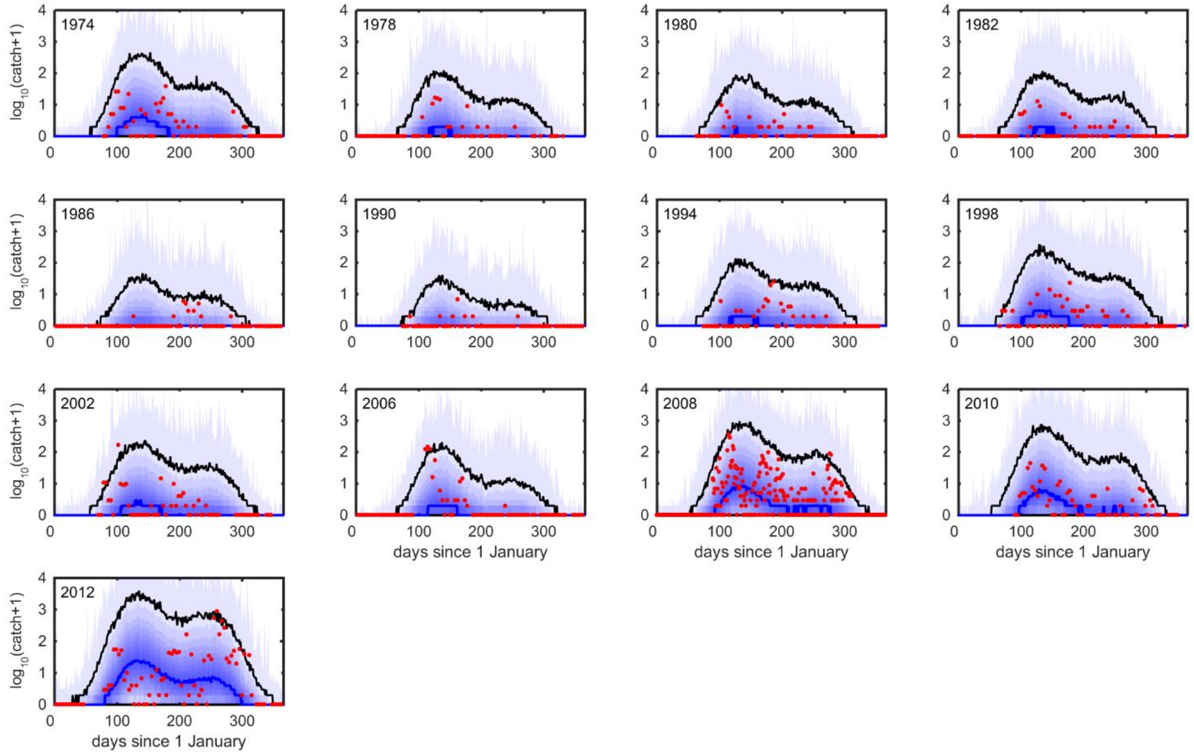
1974
1978
1980
1982
1986
1990
1994
1998
2002
2006
2008
2010
2012
log10(catch+1)
days since 1 January

Figure S25

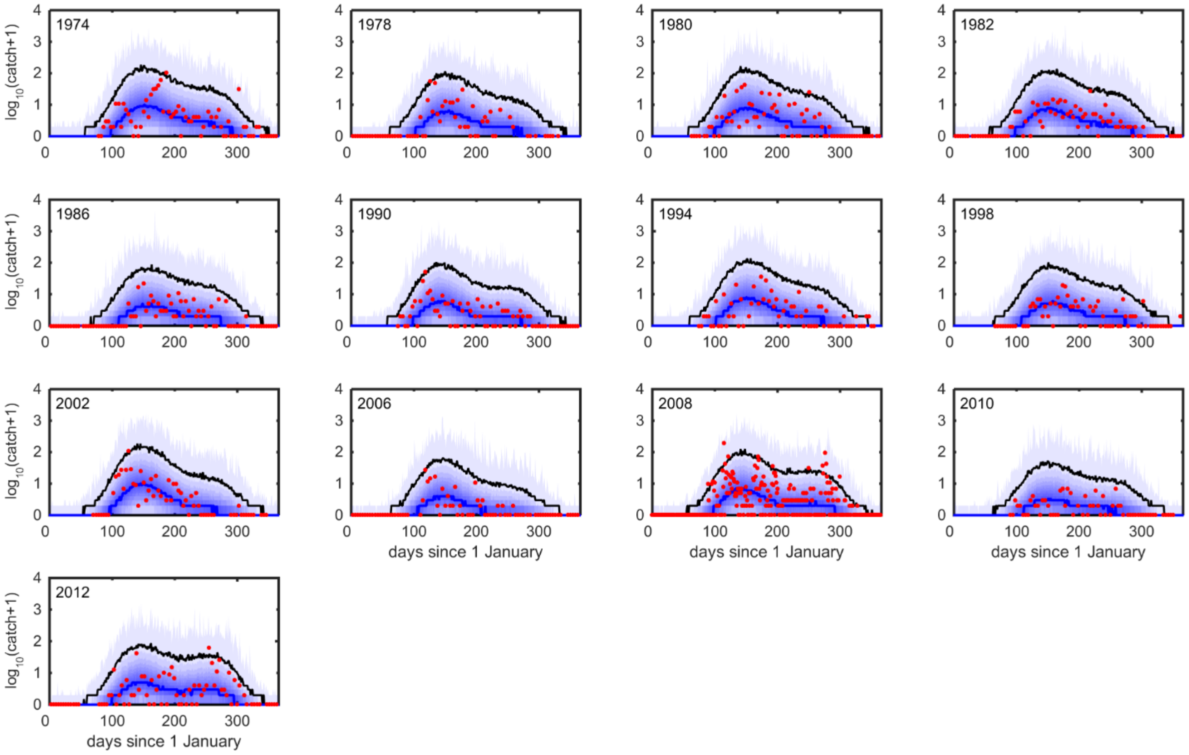
1974
1978
1980
1982
1986
1990
1994
1998
2002
2006
2008
2010
2012
log10(catch+1)
days since 1 January

Figure S26

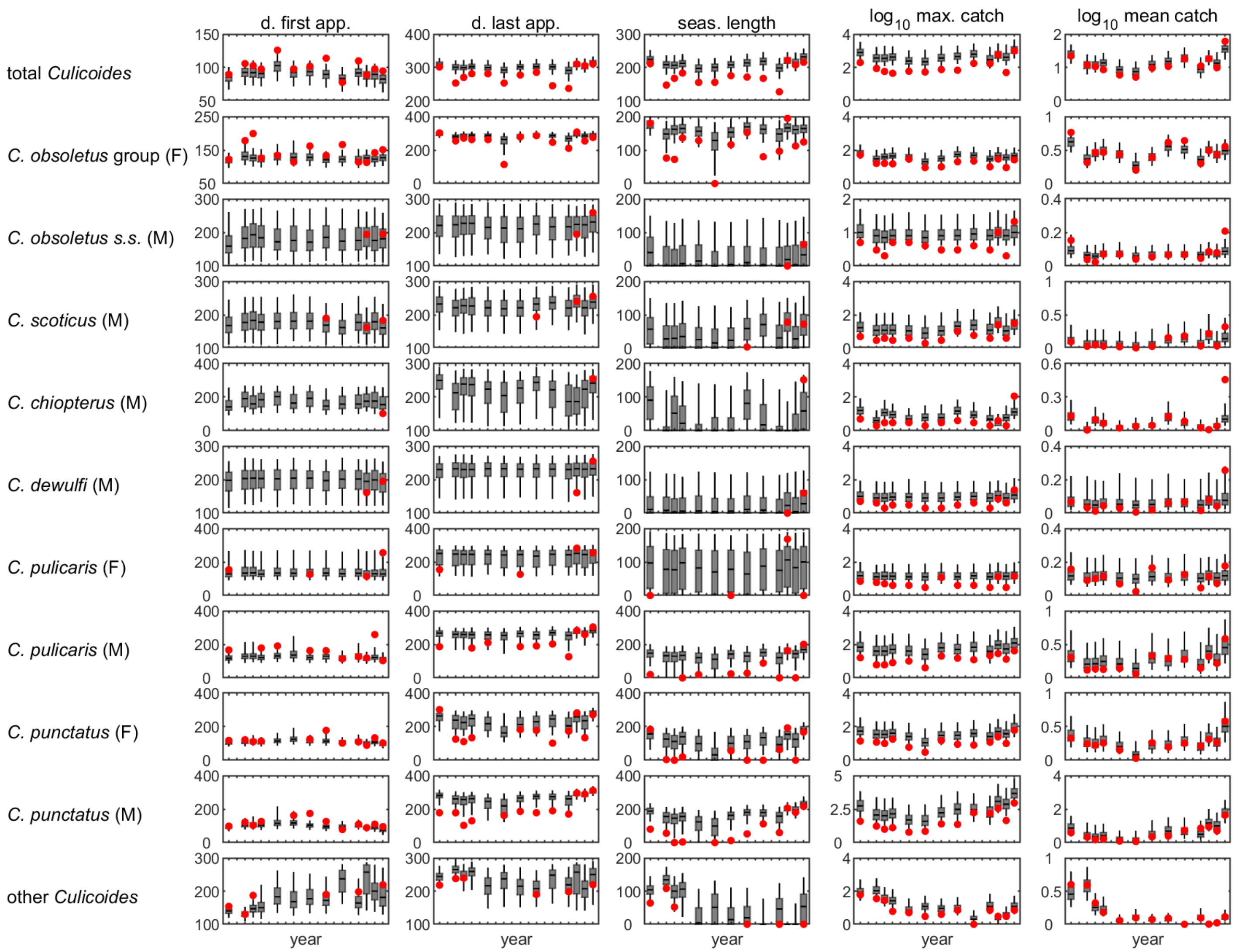

d. first app.
d. last app.
seas. length
log10 max. catch
log10 mean catch
total Culicoides
C. obsoletus group (F)
C. obsoletus s.s. (M)
C. scoticus (M)
C. chiopterus (M)
C. dewulfi (M)
C. pulicaris (F)
C. pulicaris (M)
C. punctatus (F)
C. punctatus (M)
other Culicoides
year

Figure S27

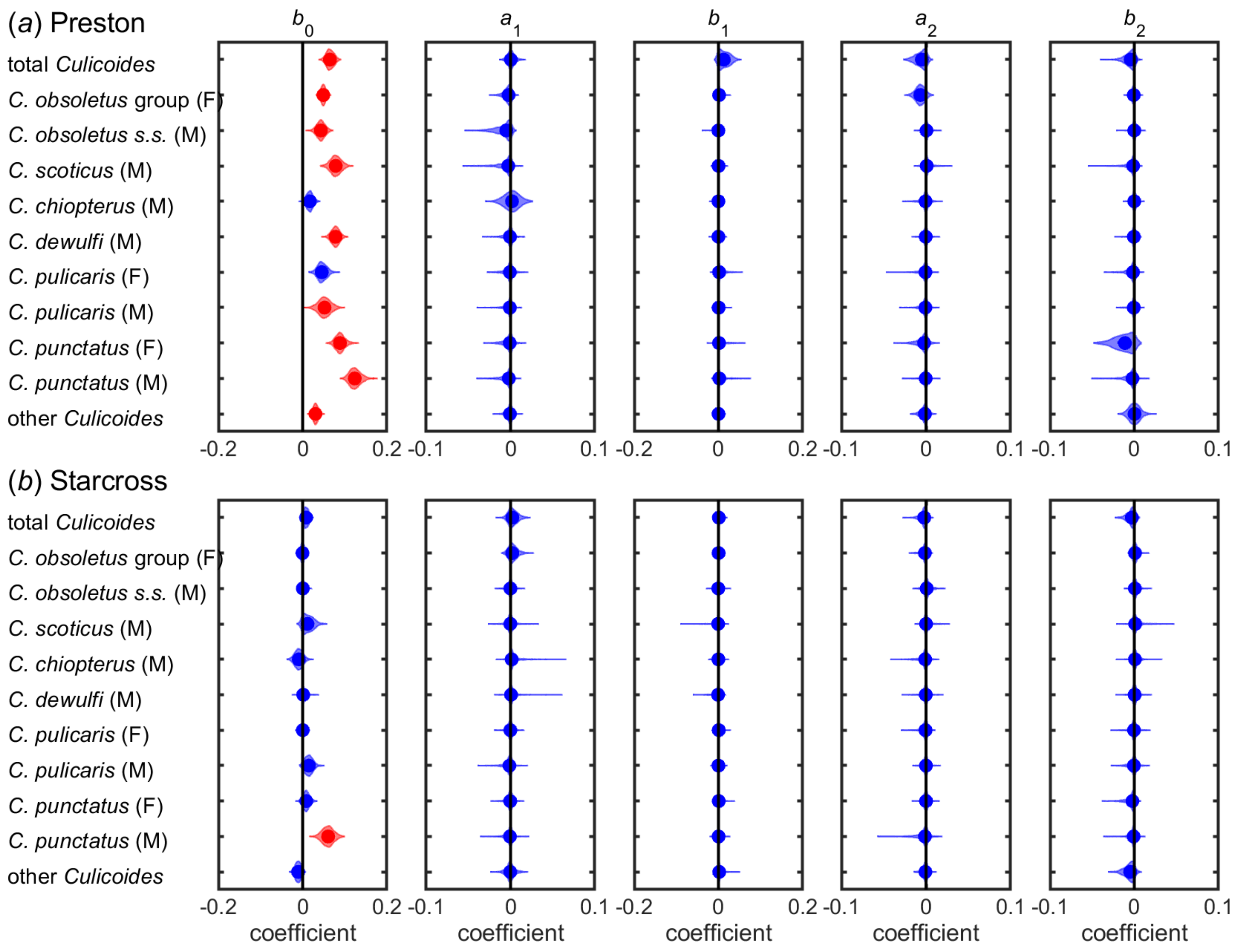
(a) Preston
b0
a1
b1
a2
b2
total Culicoides
C. obsoletus group (F)
C. obsoletus s.s. (M)
C. scoticus (M)
C. chiopterus (M)
C. dewulfi (M)
C. pulicaris (F)
C. pulicaris (M)
C. punctatus (F)
C. punctatus (M)
other Culicoides
-0.2
0
0.2
-0.1
0
0.1
(b) Starcross
coefficient

Figure S28

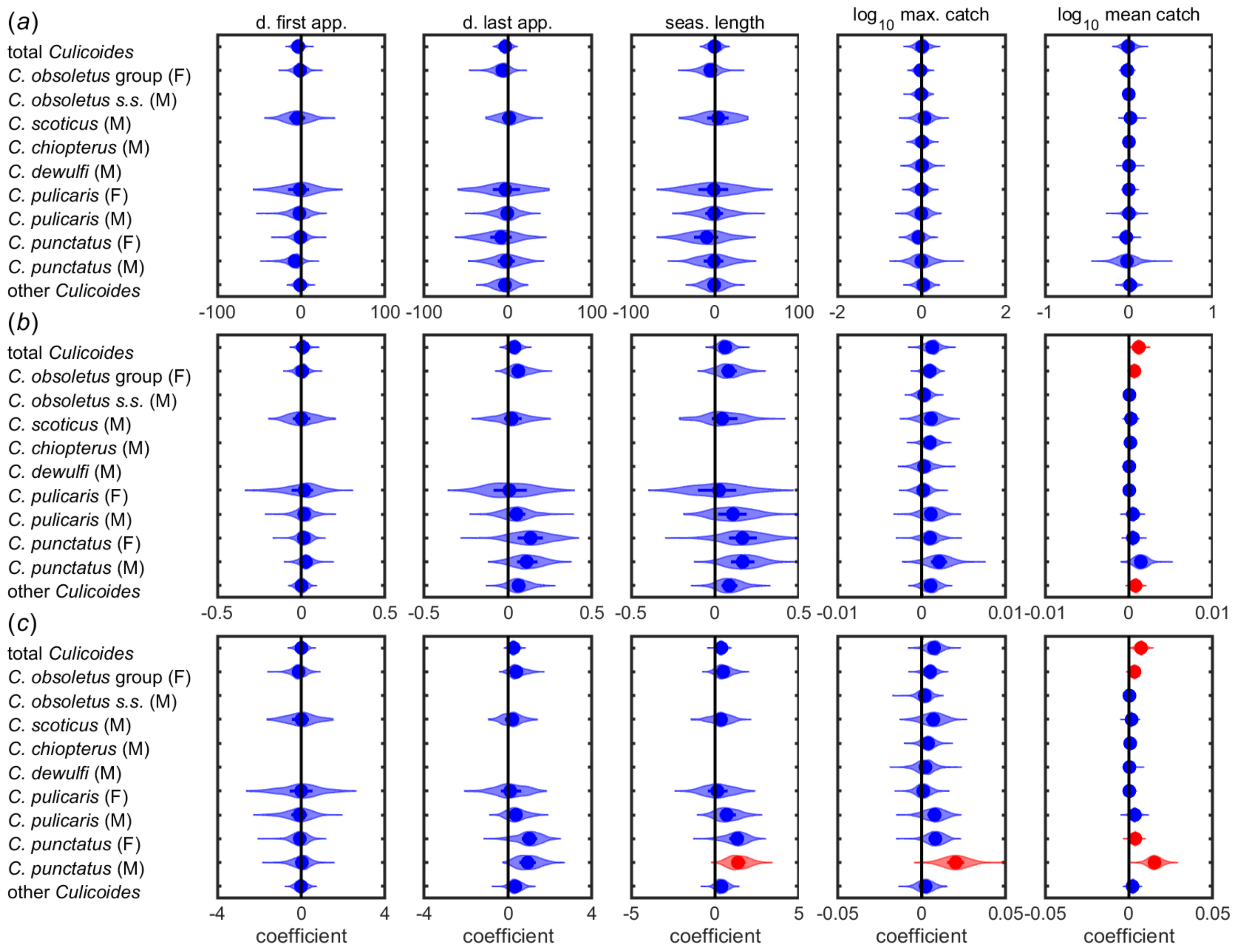
(a)
d. first app.
d. last app.
seas. length
log10 max. catch
log10 mean catch
total Culicoides
C. obsoletus group (F)
C. obsoletus s.s. (M)
C. scoticus (M)
C. chiopterus (M)
C. dewulfi (M)
C. pulicaris (F)
C. pulicaris (M)
C. punctatus (F)
C. punctatus (M)
other Culicoides
-100 0 100 -100 0 100 -100 0 100 -2 0 2 -1 0 1
(b)
-0.5 0 0.5 -0.5 0 0.5 -0.5 0 0.5 -0.01 0 0.01 -0.01 0 0.01
(c)
-4 0 4 -4 0 4 -5 0 5 -0.05 0 0.05 -0.05 0 0.05
coefficient

Figure S29

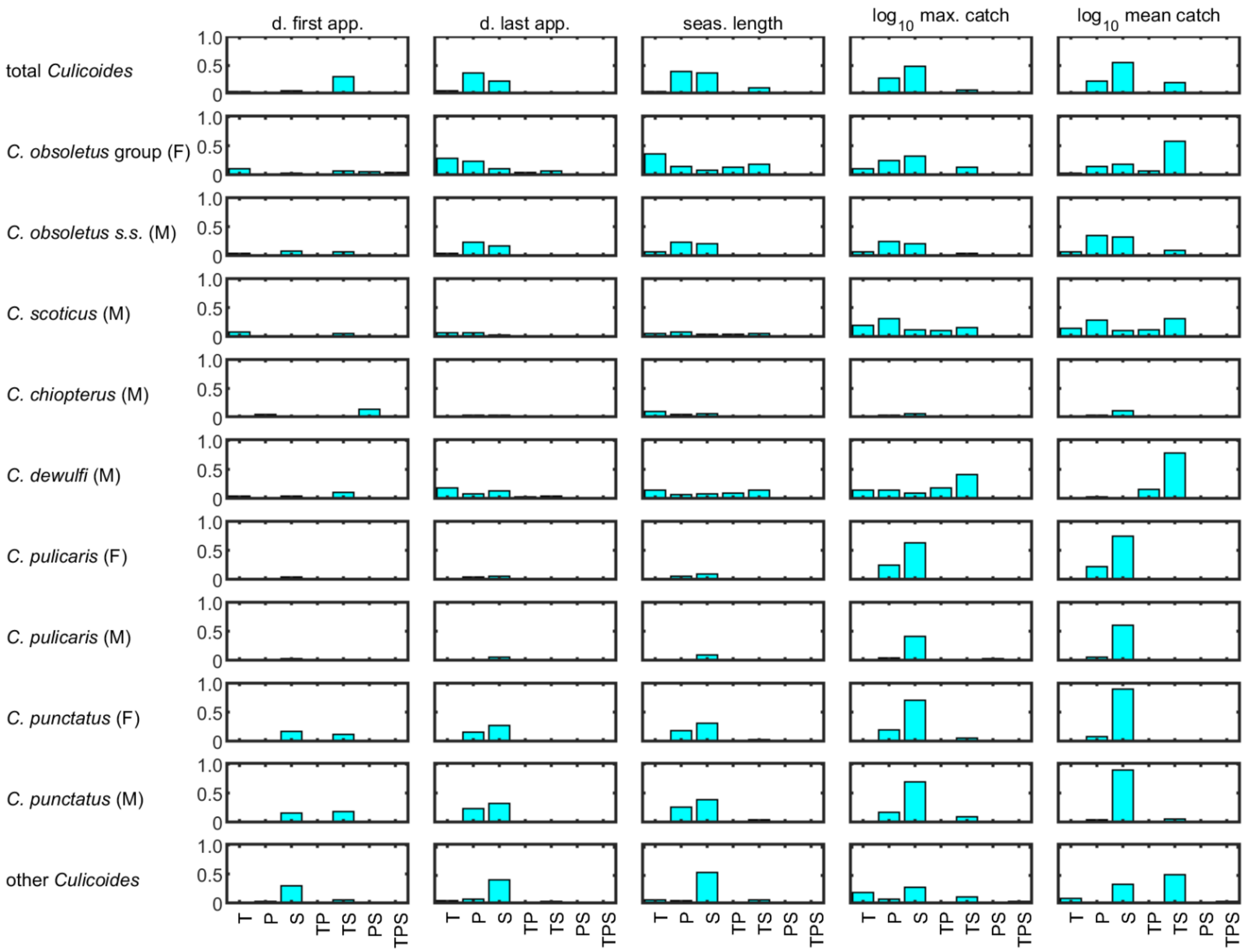
d. first app.
d. last app.
seas. length
log10 max. catch
log10 mean catch
total Culicoides
C. obsoletus group (F)
C. obsoletus s.s. (M)
C. scoticus (M)
C. chiopterus (M)
C. dewulfi (M)
C. pulicaris (F)
C. pulicaris (M)
C. punctatus (F)
C. punctatus (M)
other Culicoides
1.0
0.5
0
T
P
S
TP
TS
PS
TPS

Figure S30
